# Nutritional Status as the Key Modulator of Antioxidant Responses Induced by High Environmental Ammonia and Salinity Stress in European Sea Bass (*Dicentrarchus labrax*)

**DOI:** 10.1371/journal.pone.0135091

**Published:** 2015-08-04

**Authors:** Amit Kumar Sinha, Hamada AbdElgawad, Gaurav Zinta, Antony Franklin Dasan, Rindra Rasoloniriana, Han Asard, Ronny Blust, Gudrun De Boeck

**Affiliations:** 1 Systemic Physiological and Ecotoxicological Research, Department of Biology, University of Antwerp, Antwerp, Belgium; 2 Molecular Plant Physiology and Biotechnology group, Department of Biology, University of Antwerp, Antwerp, Belgium; 3 Department of Botany, Faculty of Science, University of Beni-Sueif, Beni-Sueif, Egypt; University of Siena, ITALY

## Abstract

Salinity fluctuation is one of the main factors affecting the overall fitness of marine fish. In addition, water borne ammonia may occur simultaneously with salinity stress. Additionally, under such stressful circumstances, fish may encounter food deprivation. The physiological and ion-osmo regulatory adaptive capacities to cope with all these stressors alone or in combination are extensively addressed in fish. To date, studies revealing the modulation of antioxidant potential as compensatory response to multiple stressors are rather lacking. Therefore, the present work evaluated the individual and combined effects of salinity challenge, ammonia toxicity and nutritional status on oxidative stress and antioxidant status in a marine teleost, European sea bass (*Dicentrarchus labrax*). Fish were acclimated to normal seawater (32 ppt), to brackish water (20 ppt and 10 ppt) and to hypo-saline water (2.5 ppt). Following acclimation to different salinities for two weeks, fish were exposed to high environmental ammonia (HEA, 20 mg/L representing 50% of 96h LC_50_ value for ammonia) for 12 h, 48 h, 84 h and 180 h, and were either fed (2% body weight) or fasted (unfed for 7 days prior to HEA exposure). Results show that in response to decreasing salinities, oxidative stress indices such as xanthine oxidase activity, levels of hydrogen peroxide (H_2_O_2_) and lipid peroxidation (malondialdehyde, MDA) increased in the hepatic tissue of fasted fish but remained unaffected in fed fish. HEA exposure at normal salinity (32 ppt) and at reduced salinities (20 ppt and 10 ppt) increased ammonia accumulation significantly (84 h–180 h) in both feeding regimes which was associated with an increment of H_2_O_2_ and MDA contents. Unlike in fasted fish, H_2_O_2_ and MDA levels in fed fish were restored to control levels (84 h–180 h); with a concomitant increase in superoxide dismutase (SOD), catalase (CAT), components of the glutathione redox cycle (reduced glutathione, glutathione peroxidase and glutathione reductase), ascorbate peroxidase (APX) activity and reduced ascorbate (ASC) content. On the contrary, fasted fish could not activate many of these protective systems and rely mainly on CAT and ASC dependent pathways as antioxidative sentinels. The present findings exemplify that in fed fish single factors and a combination of HEA exposure and reduced seawater salinities (upto 10 ppt) were insufficient to cause oxidative damage due to the highly competent antioxidant system compared to fasted fish. However, the impact of HEA exposure at a hypo-saline environment (2.5 ppt) also defied antioxidant defence system in fed fish, suggesting this combined factor is beyond the tolerance range for both feeding groups. Overall, our results indicate that the oxidative stress mediated by the experimental conditions were exacerbated during starvation, and also suggest that feed deprivation particularly at reduced seawater salinities can instigate fish more susceptible to ammonia toxicity.

## Introduction

The salinity gradient of some of the marine ecosystems typically the enclosed bays and estuaries has been gradually reducing since last few decades [1]. Ongoing global warming combined with the freshwater inputs from the rivers are the major causes resulting in the decline of salinity gradient of these natural ecosystems [2–4]. In estuarine ecosystems, salinity fluctuations is the most important environmental factor influencing physiological processes, fitness and abundance of fish species. Some euryhaline teleosts are also threatened by salinity mediated osmotic challenges as part of their migratory life cycle. Numerous studies on different fish species concerning adaptive strategies to cope with the changing environmental salinities at biochemical, physiological and molecular levels already exist [5–8]. In recent years, studies on marine teleosts have suggested that the stress caused by the changes in the ambient salinity may induce oxidative stress due to the compromised antioxidant defence system [9–12].

In natural environments, fish are challenged to various types of abiotic stresses simultaneously. For instance, hypo-osmotic stress may occur together with high environmental ammonia. High ammonia load induces a range of toxicological effects in fish, which can reduce growth rate [13,14], alter metabolism, ions and hormonal balance [14–17], and at a very high dose can even cause mortality. In general, the ionization equilibrium of (total) ammonia into toxic gaseous (NH_3_) and non-toxic ionized (NH_4_^+^) form is profoundly regulated by the salinity of the aquatic environment. Moreover, it has been documented that the high environmental ammonia (HEA) induced toxicity in several marine species can be further modulated by salinity fluctuations [15,18–22]. Similar to salinity stress, there is growing evidence that ammonia exposure can lead to oxidative stress in fish species [23–26]. However, to date there is no information on how the interactions of osmotic stress and the ammonia pollution, likely to occur together in estuaries, manipulate the oxidative damage and the response of antioxidants in fish.

Besides hypo-osmotic stress and ammonia toxicity, nutrient deprivation is a natural phenomenon which can induce stress in fish. Fish populations often encounter limited food availability in their natural habitat, and restricted feeding is often practiced in the culture system as a strategy to avoid ammonia buildup in the rearing water. Previous studies have documented that feed deprivation can elicit pro-oxidant effects and tends to deplete antioxidant stores in mammalian organs [27–30]. In contrast to mammals, limited work has been conducted regarding the consequences of starvation on oxidative stress and antioxidant mechanisms in fish [31,32].

Under normal physiological states, there is a balance between pro-oxidant production and antioxidant defences. Oxidative stress is a consequence of an imbalance in favor of pro-oxidants production as antioxidant defence systems can no longer counteract the elevated reactive oxygen species (ROS) levels [33]. Higher ROS accumulation leads to oxidation of macromolecules such as sugars, lipids, nucleic acids and cellular structures which results in metabolic dysfunction and even cell death.

ROS is scavenged by the concerted action of enzymatic and non-enzymatic antioxidant defence system.

Non-enzymatic components include glutathione (GSH), ascorbate (ASC) etc. Enzymatic components include superoxide dismutase (SOD), catalase (CAT), ascorbate peroxidase (APX), glutathione peroxidase (GPX), glutathione reductase (GR), dehydroascorbate reductase (DHAR) and glutathione-s-transferase (GST) [11,33,34].

In natural habitat, fish are often challenged to a variety of environmental stressors causing oxidative damage, and such stresses can negatively influence the fish performance by acting in isolation or in combination. Therefore, it is important to examine the interaction between different environmental variables because the biological responses to combined stress could be entirely different from the individual stress, and also the stress combination not always leads to the additive effect [35–37]. Nevertheless, the majority of the research has focused on the impact of single stressor on the oxidative stress and antioxidative defences in fish; assessment of such responses when fish are subjected to an assortment of multiple stressors such as salinity reduction, ammonia threat and starvation is rather scarce. Moreover, in our previous work with the similar experimental set up [15] we revealed physiological, metabolic, biochemical, iono-osmo regulatory and molecular mechanism in European sea bass determining their compensatory response to the individual and the combined effect of ammonia, salinity stress and feed deprivation. However, insight on the oxidative status is still lacking. Therefore, paralleling our previous study [15], this work aims to extend our understanding on how the antioxidant defence mechanism are modulated in fish resulting from the oxidative stress induced by the combined effects of ammonia pollution and feed deprivation under different salinity gradients.

European sea bass (*Dicentrarchus labrax* L.) is one of the most preferred fish species in Europe for aquaculture which also possess high commercial and ecological value. This fish species seasonally migrate between open sea and estuaries or lagoons, and thus often challenged with hypo-saline environment. Therefore, in the present study, we used juveniles of European sea bass as a test organism to examine how this species manipulates its antioxidative compensatory responses in order to cope with oxidative stress when introduced to different stressors such as low ambient salinity, high environmental ammonia and feed deprivation simultaneously. Usually, studies focusing on the impact of environmental stressors investigate the responses at a single time point. Temporal analyses conducted in the present study provide a better understanding on the impact of stress effects, and provide view on early and late responses.

Liver is a vital organ and plays a major role in homeostasis. It is metabolically active, consequently, expected to possess higher ROS production rates. It is therefore the preferred organ for assessing the status of oxidative stress and antioxidant defences in living organisms, and is also highly relevant for biomonitoring studies [26,38–41]. Overall, we hypothesized that sea bass would be adversely affected by salinity challenge and HEA, and the oxidative stress induced by hypo-osmotic environments would be exacerbated when fish are confronted with ammonia exposure. We also anticipate that feeding would activate the antioxidant compensatory responses for effectively eliminating the excess ROS production and minimizing the cellular damage. To test these hypotheses, we investigate the effects of multiple-stressors encompassing high environmental ammonia (20 mg/L, represents 50% of 96h LC_50_ value expressed as total ammonia at pH 8.1; Person-Le Ruyet et al. [42]) and periods of feed deprivation in hepatic tissue of European sea bass during experimental salinity stress (i.e., acclimation to the different salinities- 32, 20,10 and 2.5 ppt) on (i) the intensity of oxidative damage through the examination of oxidative stress indicators e.g. H_2_O_2_, malondialdehyde (MDA) and xanthine oxidase (XO), (ii) the kinetics of antioxidant defence system by the analysis of antioxidant molecules (GSH and ASC) and enzymes (SOD, CAT, APX, DHAR, GPX, GR and GST).

## Materials and Methods

### Experimental system and animals

European sea bass (*Dicentrarchus labrax*) juveniles (14–18 g) were obtained from Ecloserie Marine (Gravelines, France) and transferred to the University of Antwerp. Fish were maintained in 1000 L tanks, filled with artificial seawater (Meersalz Professional Salt, 32 ppt salt). Thereafter, a total of 480 fish were distributed into twenty four 200 L tanks (n = 20 per tank; 32 ppt) equipped with a recirculating water supply in a climate chamber where temperature was adjusted at 17±1°C and photoperiod was 12 h light and 12 h dark. Fish were acclimated to the above mentioned constant salinity, temperature and photoperiod for one month prior to the experiment and were fed with commercial pellets (Skretting, Boxmeer, The Netherlands) at a rate of 2% on their wet body weight/day. Water quality was ensured through an additional bio-filter containing wadding, activated charcoal and lava stones. During the ammonia exposure, charcoal and lava stones were removed from the filter to prevent ammonia absorption in the filter. Similarly, they were removed from control tanks as well. All animal experiments were approved by the local ethics committee, University of Antwerp (Permit Number: LA1100134), and conducted according to the guidelines of the Federation of European Laboratory Animal Science Associations.

### Hypo-osmotic stress: fish acclimation to lowered seawater salinities

Fish in the tanks were progressively acclimated to three experimental salinities: 20 ppt (~ 500 mOsm/Kg, pH 8.17; 6 tanks); 10 ppt (~ 249 mOsm/Kg, pH 8.10; 6 tanks) and 2.5 ppt (~ 69 mOsm/Kg, pH 7.87; 6 tanks). Fish in the remaining 6 tanks were maintained at normal seawater salinity 32 ppt (~ 800 mOsm/Kg, pH 8.2). The salinities of 20 ppt and 10 ppt correspond to brackish water, while 2.5 ppt characterizes hyposaline water. Changes in salinity were progressed by reducing the salinity by 5%, each three days until the desired salinity was reached. Experimental salinities were adjusted by diluting artificial seawater with filtered freshwater, and salinity was measured using a hand-held refractometer. Each experimental group was acclimatized to the desired salinity for 2 weeks and was fed daily at a rate of 2% of their wet body weight.

### Experimental groups and ammonia exposure

After being acclimatized at the respective salinities for 2 weeks, feeding was withheld in three of the tanks, one of each of the salinity regimes. Fasted fish groups were kept unfed 7 days prior to the ammonia exposure, while feeding (2% body weight/day) was continued in the respective parallel tanks. Feeding was adjusted based on the weight and the number of fish remaining in the tank after each sampling period.

In brief, the experimental set up for each of the salinity group consists of four categories: (1) ammonia unexposed (control) fed fish (1 tank), (2) ammonia exposed fed fish (2 tanks), (3) ammonia unexposed (control) starved fish (1 tank) and, (4) ammonia exposed starved fish (2 tanks). Each exposure tank was spiked with the required amount of an NH_4_HCO_3_ stock solution (Sigma, Germany). A constant concentration of 20 ± 0.18 mg/L of (total) ammonia was maintained throughout the experiment. Ammonia concentrations were measured (using the salicylate-hypochlorite method, Verdouw et al. [43]) each 6 h after the onset of treatment and the concentration of ammonia in the tank was maintained by adding the calculated amount of the NH_4_HCO_3_ solution. To avoid the microbial breakdown of test chemical and build-up of other waste products, 40–60% of the water was discarded twice a week and replaced with fresh water containing the respective amount of ammonia. The salinity was tested and controlled daily by adding clean water (of the appropriate salinity). Water pH was monitored throughout the experimental period using a pH electrode (Hamilton Bonaduz AG, Metrohm) and was maintained constantly at the respective control levels.

Ammonia exposed groups for both fed and fasted groups acclimated to experimental salinities were sampled after 12 h, 48 h, 84 h and 180 h. Following each exposure time, four fish for each feeding groups were sampled from each of their respective two tanks. Control groups (no HEA) were set up in parallel to the first (12 h) and the last sampling period (180 h) and were sampled in an identical way as for the exposure groups.

### Sampling procedure

For sampling, fish (n = 8) were removed from tanks and anesthetized using an overdose of neutralized MS222 (pH 8.0, ethyl 3-aminobenzoate methane-sulfonic acid, 1 g/L, Acros Organics, Geel, Belgium). Fish were dissected on ice; liver were removed, snap frozen in liquid nitrogen, and stored at -80°C for determination of ammonia content, oxidative stress parameters, antioxidant molecules and enzymes. Ammonia content was determined according to Wright et al. [44] using an enzymatic kit (R-Biopharm AG, Darmstadt, Germany).

### Quantification of oxidative stress indices

MDA content, an end product of lipid peroxidation was assayed according to Hodges et al. [45]. 50 mg of liver tissue was homogenized in 1 mL of 80% ethanol using MagNALyser (Roche, Vilvoorde, Belgium) and reacted with thiobarbituric acid to produce pinkish red chromogen thiobarbituric acid-malondialdehyde (TBA-MDA). Absorbance at 440, 532 and 600 nm was measured using in a micro-plate reader. MDA content was calculated and expressed as nmol/g wet tissue.

For quantification of H_2_O_2_, 50 mg of liver tissues were homogenized in 50 mM phosphate buffer (pH 6.5) and was measured by the FOX1(ferrous oxidation-xylenol orange) method [46].

### Determination of Antioxidant molecules content

Reduced ascorbate (ASC) and reduced glutathione (GSH) were quantified by high-performance liquid chromatography (Reversed-Phase HPLC of Shimadzu, Hai Zhonglu, Shanghai) following the methodology described by Sinha et al. [26]. Liver tissue was homogenized under liquid nitrogen using a mortar and pestle. The resulting powder was thawed on ice in a 6% metaphosphoric acid (MPA) solution (0.5 ml MPA/100 mg wet tissue). After homogenization, the samples were centrifuged at 14,000 rpm for 12 min. 100 μL of the supernatant was added to 300μL eluens (2 mM KCl, pH 2.5). Antioxidants were separated on HPLC by injecting 10μL onto a Polaris C18-A column with a 1 mL/min flow rate.

The concentrations of reduced glutathione (GSH), total glutathione (tGSH), reduced ascorbate (ASC) and total ascorbate (tASC) were calculated using a standard curve created by known concentrations of GSH and ASC and expressed in terms of nmol/g wet weight. The standards were prepared freshly before use (Peak Asc: 242 nm, peak GSH: 196 nm; Retention time of ASC was 1.7–1.8 min and of GSH 2.2–2.3 min). Oxidized glutathione (GSSH) content was calculated as the difference between the content of tGSH and GSH. Similarly, the content of oxidized ascorbate (dehydroascorbate, DHA) represents the difference between tASC and ASC.

### Enzyme assay

Xanthine oxidase (XO), Superoxide dismutase (SOD), catalase (CAT), glutathione peroxidadase (GPX), ascorbate peroxidase (APX), glutathione reductase (GR), glutathione-s- transferase (GST) and dehydroascorbate reductase (DHAR) were determined from the homogenate prepared in 1 mL of 50 mM potassium phosphate buffer (pH 7.0) containing 10% polyvinyl pyrrolidone (PVP), 0.25% Triton X-100, 0.001 M polymethyl sulfonyl fluoride (PMSF) and 0.001 M ascorbate using MagNALyser. SOD activity was determined according to Dhindsa et al. [47] by measuring the inhibition of NBT (nitroblue tetrazolium) reduction at 560 nm. CAT activity was assayed according to the procedure of Aebi [48] by monitoring the rate of decomposition of H_2_O_2_ at 240 nm. The NBT assay was carried out to determine the XO activity by following the methodology of Ozer et al. [49], and the absorbance was recorded at 575 nm. APX, DHAR and GR activities were measured by the method of Murshed et al. [50]. GST enzyme activity was calculated by measuring the conjugation of GSH with excess 1-chloro-2,4-dinitrobenzene (CDNB) at 340 nm [51]. GPX activity was assayed by measuring the decrease in NADPH absorbance measured at 340 nm [52]. All activity measurements were scaled down for semi-high throughput using a micro-plate reader (Synergy Mx, Biotek Instruments Inc., Vermont, USA), and optimized to obtain linear time and protein-concentration dependence. The soluble protein content was estimated according to Lowry et al. [53].

### Statistical analysis

All data have been presented as mean values ± standard error (S.E.). For comparisons between different experimental groups a one-way analysis of variance (ANOVA) was performed followed by the least significant difference (LSD) test. Student’s two-tailed t-test was used for single comparisons. Main effects of salinity challenge, ammonia exposure and feeding status and their interactions were analyzed by three-way ANOVA. Pearson correlation was performed to show the relationship among various variables.The data were analyzed by Statistical Package for the Social Sciences (SPSS) version 20.0. A probability level of 0.05 was used for rejection of the null hypothesis. Principal component analysis (PCA) was performed by using OriginLab 9 software (OriginLab, Northampton, MA, U.S.A.). All measured parameters were subjected to PCA to investigate the overall effect of stressors on the oxidative status and antioxidative response in European sea bass. The standardized scores of the first two components which explained the highest variation were used to make biplots.

No significant differences were found between any of the control values at 12 h and 180 h. Therefore, pooled controls for each experimental group are shown for clarity of the figures.

## Results

### Ammonia level

**Fig 1:**
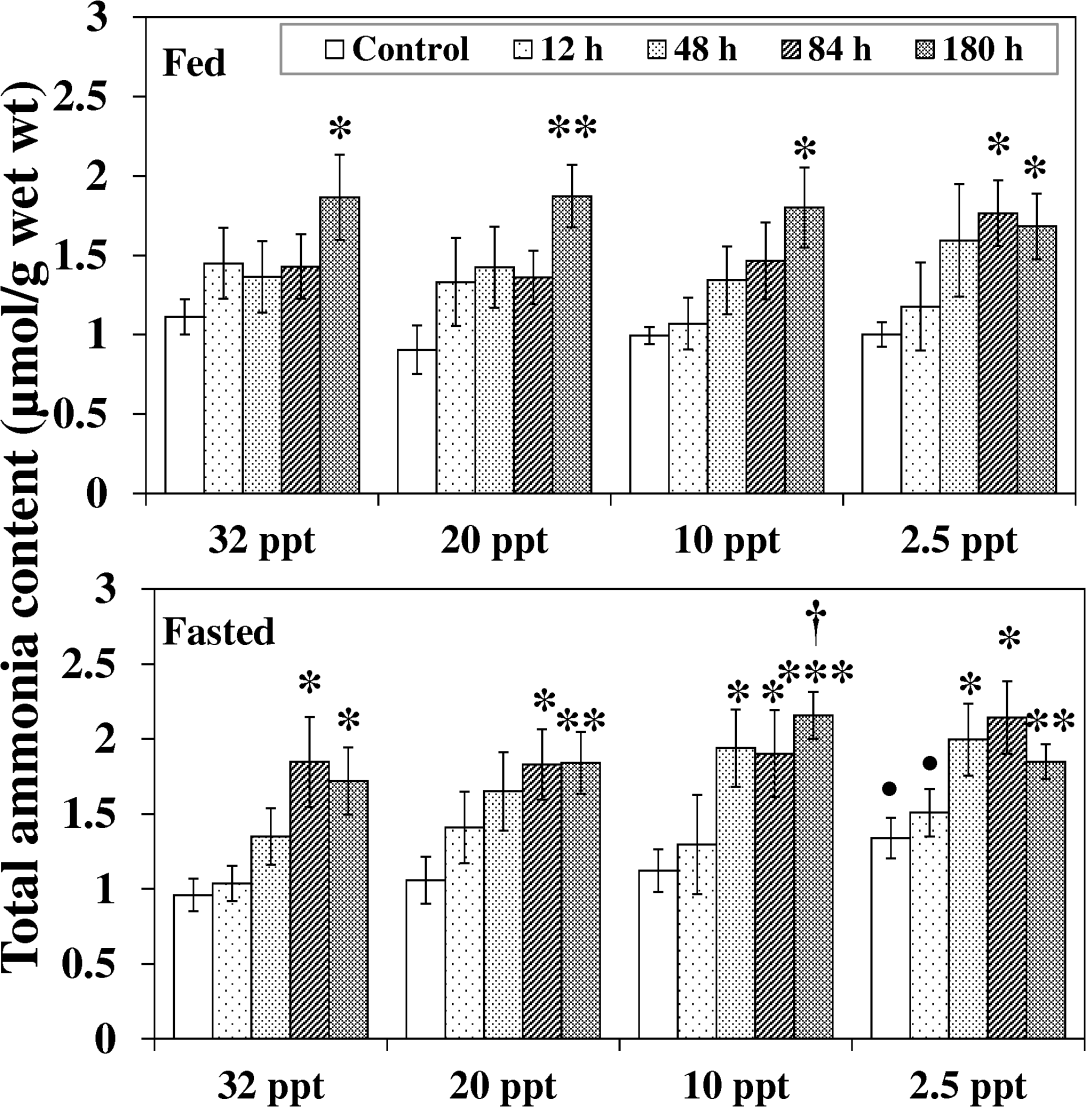
Ammonia accumulation. Ammonia accumulation (μmol/g) in liver of fed and fasted fish during acclimation to different salinities and exposure to HEA. Values are mean ± S.E. Asterisk (*) indicates a significant difference between the ammonia exposed fish and its respective control at each salinity (*P < 0.05; **P < 0.01; ***P < 0.001), bullet (•) indicates a significant difference between experimental salinities (20 ppt -2.5 ppt) and the 32 ppt-acclimated fish at the same sampling period (^•^ *P* < 0.05), dagger (†) denotes the significant difference between fed fish and its respective fasted fish counterpart (^†^ *P* < 0.05).

Internal ammonia load is an indicator of ammonia induced toxicity. Comparison among control groups of seawater (32 ppt), brackish water (20, 10 ppt) and hyposaline water (2.5 ppt) acclimated fish shows that the ammonia accumulation in hepatic tissue of fasted fish at 2.5 ppt was 40% higher (*P* < 0.05) corresponding to the respective 32 ppt group (Fig 1).

The effect of HEA on 32 ppt-20 ppt fasted fish was pronounced (*P* < 0.05) from 84 h onwards: ammonia accumulation augmented considerably compared to the respective control group and persisted until the end of the exposure period (Fig 1). Such increments became significant from 48 h HEA onwards at lower salinities of 10 ppt and 2.5 ppt. Similar patterns in response to HEA exposure were observed for fed fish, but the increase was delayed and became significant at 84 h-180 h. The effect of nutritional status on ammonia accumulation during HEA exposure was seen only at 10 ppt: ammonia level in fasted fish was 21% higher (*P* < 0.05) during 180 h HEA compared to their fed counterpart.

### Oxidative indices

#### H_2_O_2_ and MDA content

**Fig 2:**
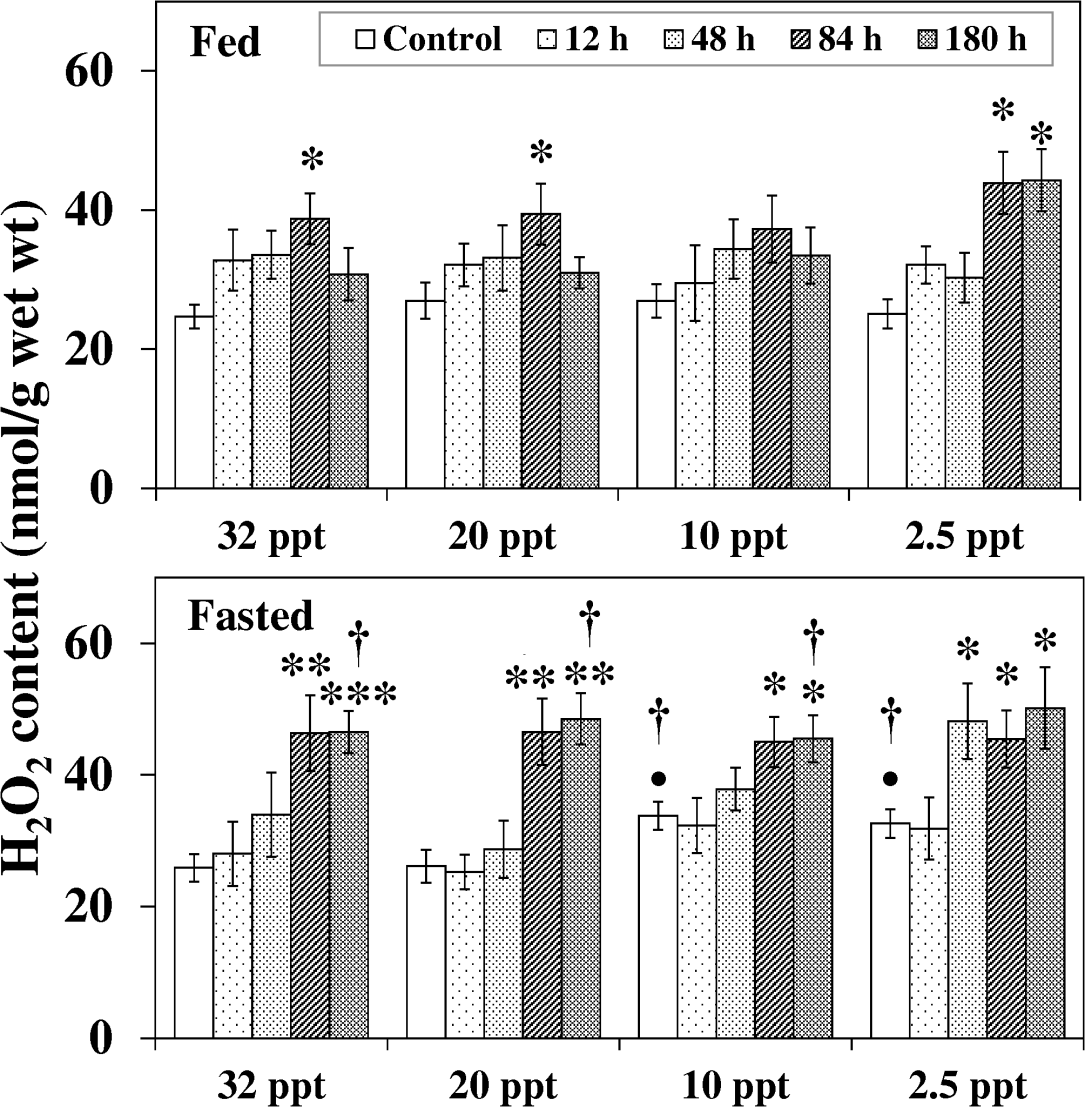
Hydrogen peroxide content. Hydrogen peroxide (H_2_O_2_) content (nmol/g) in liver of fed and fasted fish during acclimation to different salinities and exposure to HEA. Values are mean ± S.E. Asterisk (*) indicates a significant difference between the ammonia exposed fish and its respective control at each salinity (*P < 0.05; **P < 0.01; ***P < 0.001), bullet (•) indicates a significant difference between experimental salinities (20 ppt -2.5 ppt) and the 32 ppt-acclimated fish at the same sampling period (^•^ *P* < 0.05), dagger (†) denotes the significant difference between fed fish and its respective fasted fish counterpart (^†^ *P* < 0.05).

Salinity reduction itself had a profound effect on H_2_O_2_ level in fasted fish only (Fig 2). Compared to the 32 ppt control, H_2_O_2_ content was notably elevated in fasted fish acclimated to10 ppt and 2.5 ppt with a 31% (*P* < 0.05) and 27% (*P* < 0.05) increment respectively. These H_2_O_2_ levels were also significantly higher than in the corresponding fed fish.

During exposure to HEA at 32 ppt-10 ppt, H_2_O_2_ content increased significantly in fasted fish from 84 h exposure onwards. In fed fish, a transient increase (*P* < 0.05) at 84 h was followed by a recovery thereafter. The effect of HEA was more pronounced at the lowest salinity (2.5 ppt): the increment in fasted fish was seen from 48 h onwards, while in fed fish the H_2_O_2_ level was elevated (*P* < 0.05) during 84 h–180 h. The effect of fasting was seen at 180 h HEA exposure in the salinity range of 32 ppt -10 ppt: H_2_O_2_ levels in fasted fish augmented significantly compared to their fed counterpart.

**Fig 3:**
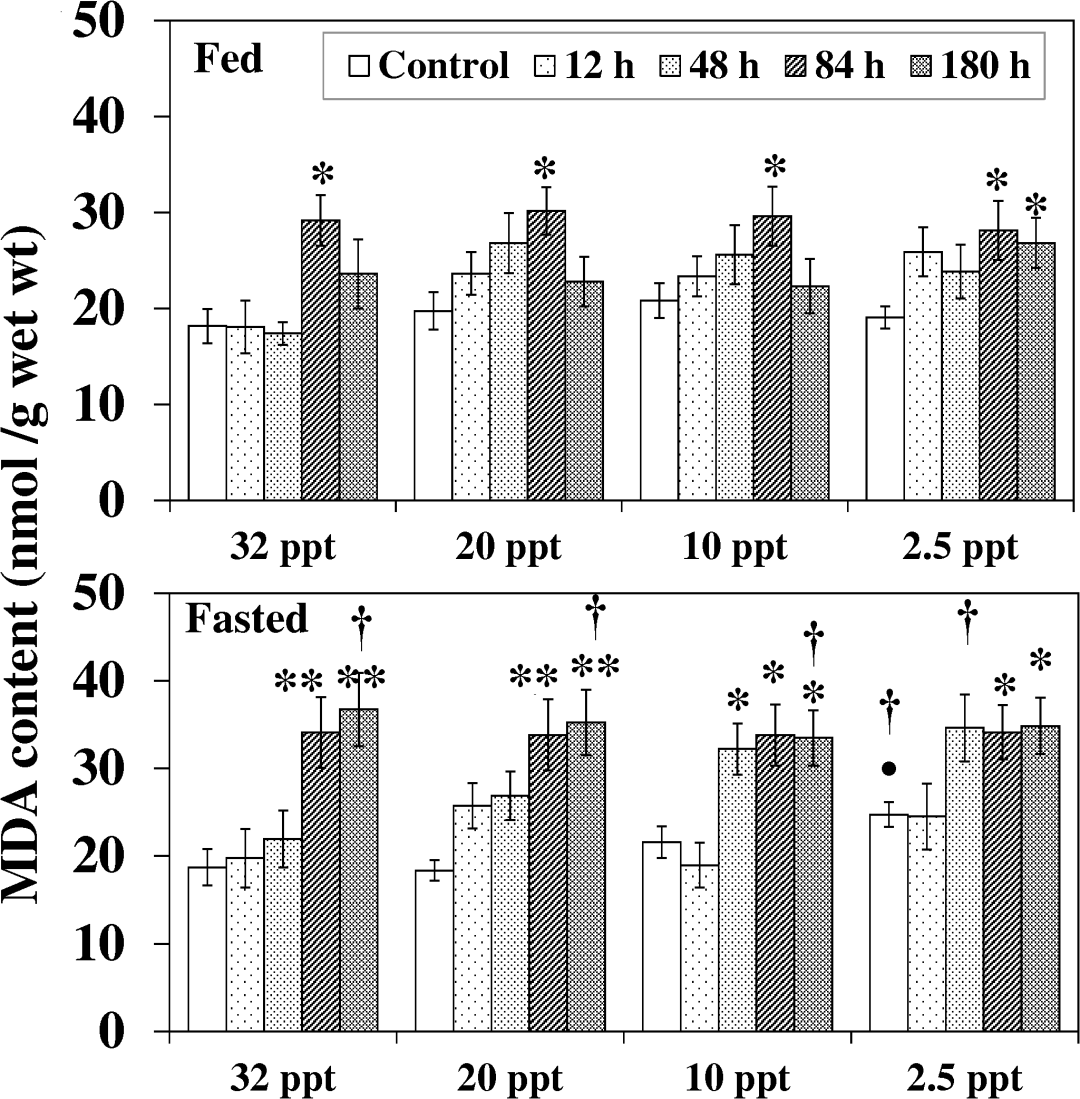
Malondialdehyde content. Malondialdehyde (MDA) content (nmol/g) in liver of fed and fasted fish during acclimation to different salinities and exposure to HEA. Values are mean ± S.E. Asterisk (*) indicates a significant difference between the ammonia exposed fish and its respective control at each salinity (*P < 0.05; **P < 0.01), bullet (•) indicates a significant difference between experimental salinities (20 ppt -2.5 ppt) and the 32 ppt-acclimated fish at the same sampling period (^•^ *P* < 0.05), dagger (†) denotes the significant difference between fed fish and its respective fasted fish counterpart (^†^ *P* < 0.05).

MDA content in both fed and fasted fish under salinity stress and ammonia exposure followed almost the same pattern as the H_2_O_2_ levels, and differences were found between the fed and fasted counterparts as illustrate in Fig 3.

#### Xanthine oxidase (XO) activity

**Fig 4:**
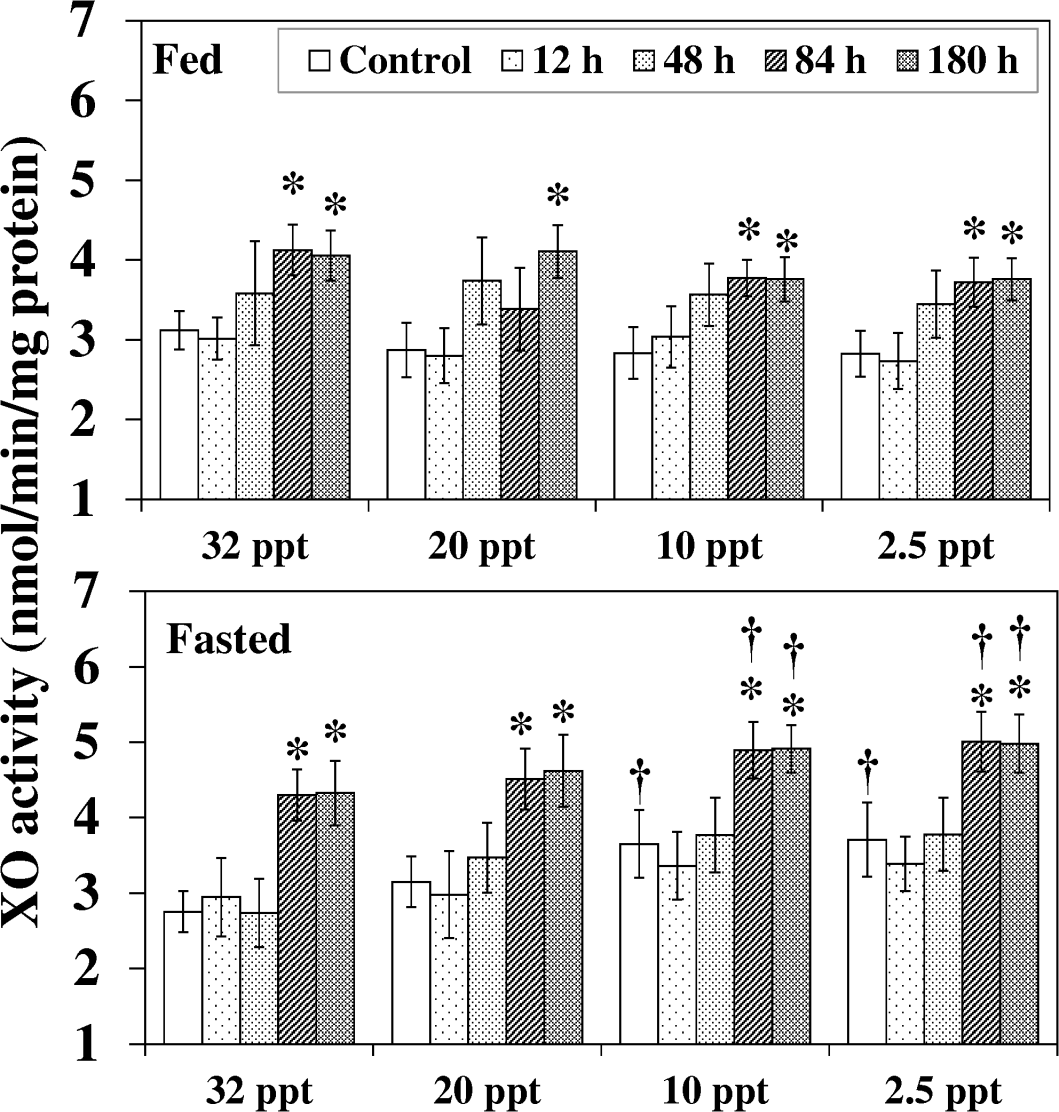
Xanthine oxidase activity. Xanthine oxidase (XO) activity (nmol/min/mg protein) in liver of fed and fasted fish during acclimation to different salinities and exposure to HEA. Values are mean ± S.E. Asterisk (*) indicates a significant difference between the ammonia exposed fish and its respective control at each salinity (*P < 0.05), dagger (†) denotes the significant difference between fed fish and its respective fasted fish counterpart (^†^ *P* < 0.05).

XO activity in hepatic tissue was affected by HEA and nutritional status (Fig 4). HEA exposure (84 h-180 h) for both fed and fasted fish at each of the experimental salinities resulted in an increase in XO activity. At 10 ppt and 2.5 ppt, the augmentation in control and HEA exposed (84 h and 180h) fasted fish were also significantly higher than in the parallel fed group. In addition, at these two salinities (10 ppt and 2.5 ppt) the activity in control fasted fish also prevailed the corresponding fed fish by 29% (*P* < 0.05) and 32% (*P* < 0.05) respectively.

### Antioxidant defence system

#### ROS scavenging enzymes

**Fig 5:**
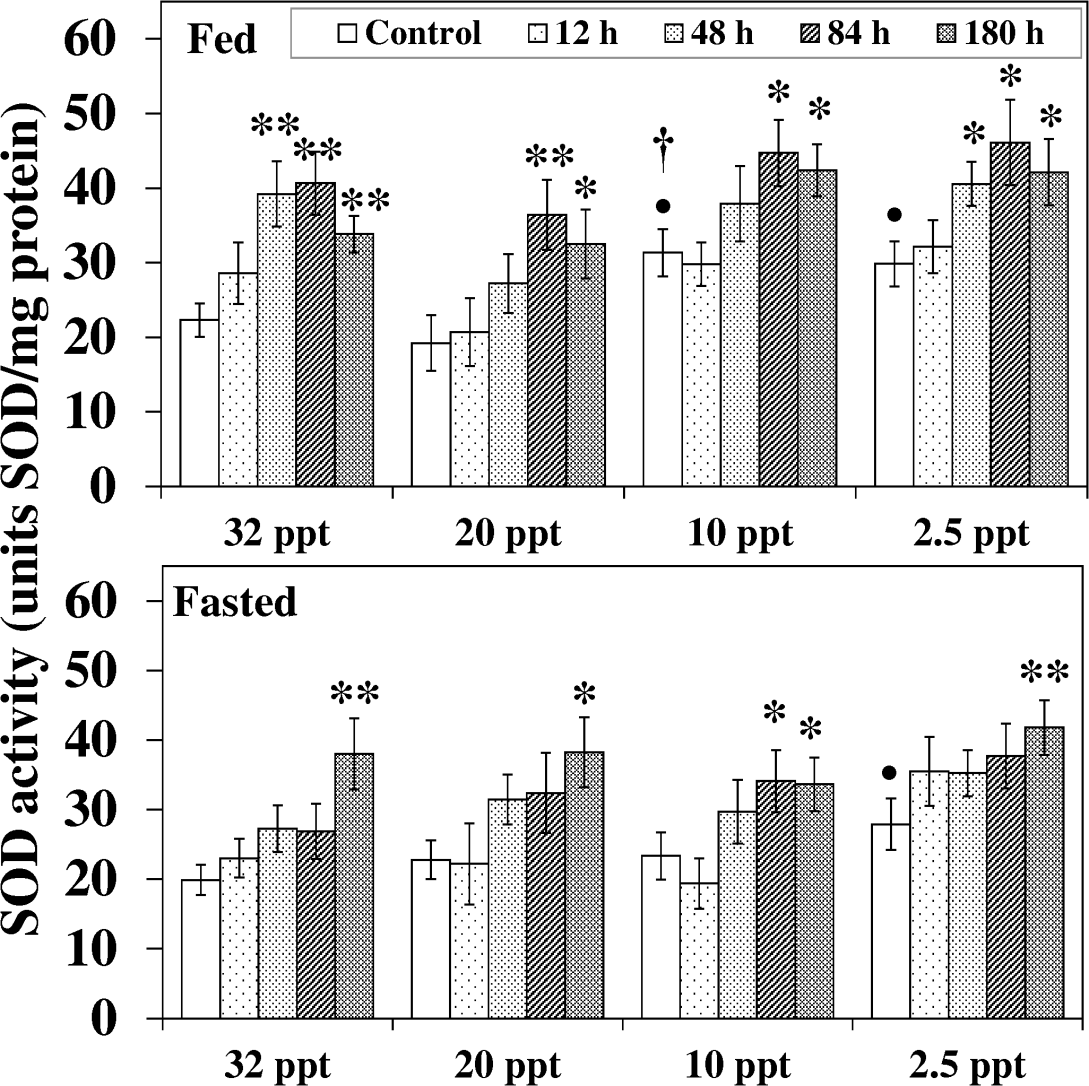
Superoxide dismutase activity. Superoxide dismutase (SOD) activity (units SOD/mg protein) in liver of fed and fasted fish during acclimation to different salinities and exposure to HEA. Values are mean ± S.E. Asterisk (*) indicates a significant difference between the ammonia exposed fish and its respective control at each salinity (*P < 0.05; **P < 0.01), bullet (•) indicates a significant difference between experimental salinities (20 ppt -2.5 ppt) and the 32 ppt-acclimated fish at the same sampling period (^•^ *P* < 0.05), dagger (†) denotes the significant difference between fed fish and its respective fasted fish counterpart (^†^ *P* < 0.05).

SOD activity in both feeding groups augmented in response to salinity reduction alone (Fig 5). A significant increment of 41% and 35% respectively was noted for 10 ppt and 2.5 ppt control fed fish relative to the 32 ppt acclimated fish while in fasted fish, a momentous increment (41%, *P* < 0.05) was seen at 2.5 ppt. HEA exposure in 32 ppt-2.5 ppt acclimated fed and fasted fish induced an increase in SOD activity (Fig 5). In fed fish, a significant increment was recorded from 48 h-84 h onwards; such increment was postponed in fasted fish and became prominent only at the last exposure period (except for 10 ppt). The effect of feeding during HEA exposure was seen at the lower salinity (10 ppt); SOD activity in control fed fish augmented by 35% (*P* < 0.05) compared to their fasted counterpart.

**Fig 6:**
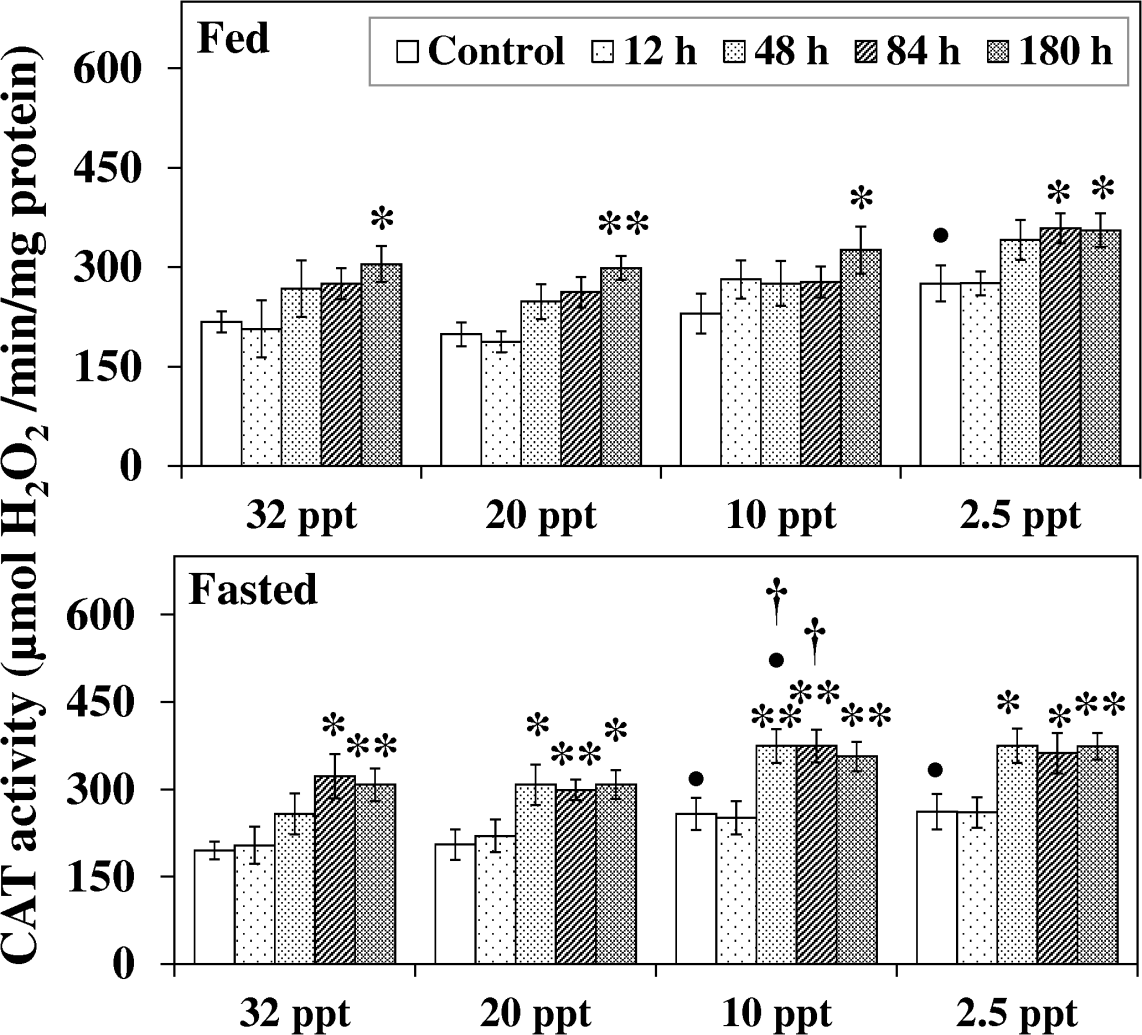
Catalase activity. Catalase (CAT) activity (mmol H_2_O_2_/min/mg protein) in liver of fed and fasted fish during acclimation to different salinities and exposure to HEA. Values are mean ± S.E. Asterisk (*) indicates a significant difference between the ammonia exposed fish and its respective control at each salinity (*P < 0.05; **P < 0.01), bullet (•) indicates a significant difference between experimental salinities (20 ppt -2.5 ppt) and the 32 ppt-acclimated fish at the same sampling period (^•^ *P* < 0.05), dagger (†) denotes the significant difference between fed fish and its respective fasted fish counterpart (^†^ *P* < 0.05).

The effect of salinity on CAT activity had more profound effects in fasted fish. The activity in fasted fish rose considerably at 10 ppt and 2.5 ppt whereas in fed fish the increment was seen only at the lowest salinity (2.5 ppt) (Fig 6). Though only a modest stimulation of SOD activity was noted for HEA exposed fasted fish at experimental salinities, a prominent elevation (*P* < 0.05–0.01) in CAT activity was recorded from 48 h-84 h onwards (Fig 6). On the contrary, for HEA exposed fed fish a significant increase in CAT activity was observed primarily at the last exposure period. Also, at 10 ppt the increments in 48 h and 84 h HEA exposed fasted fish were significantly higher than in the respective fed counterparts.

**Fig 7:**
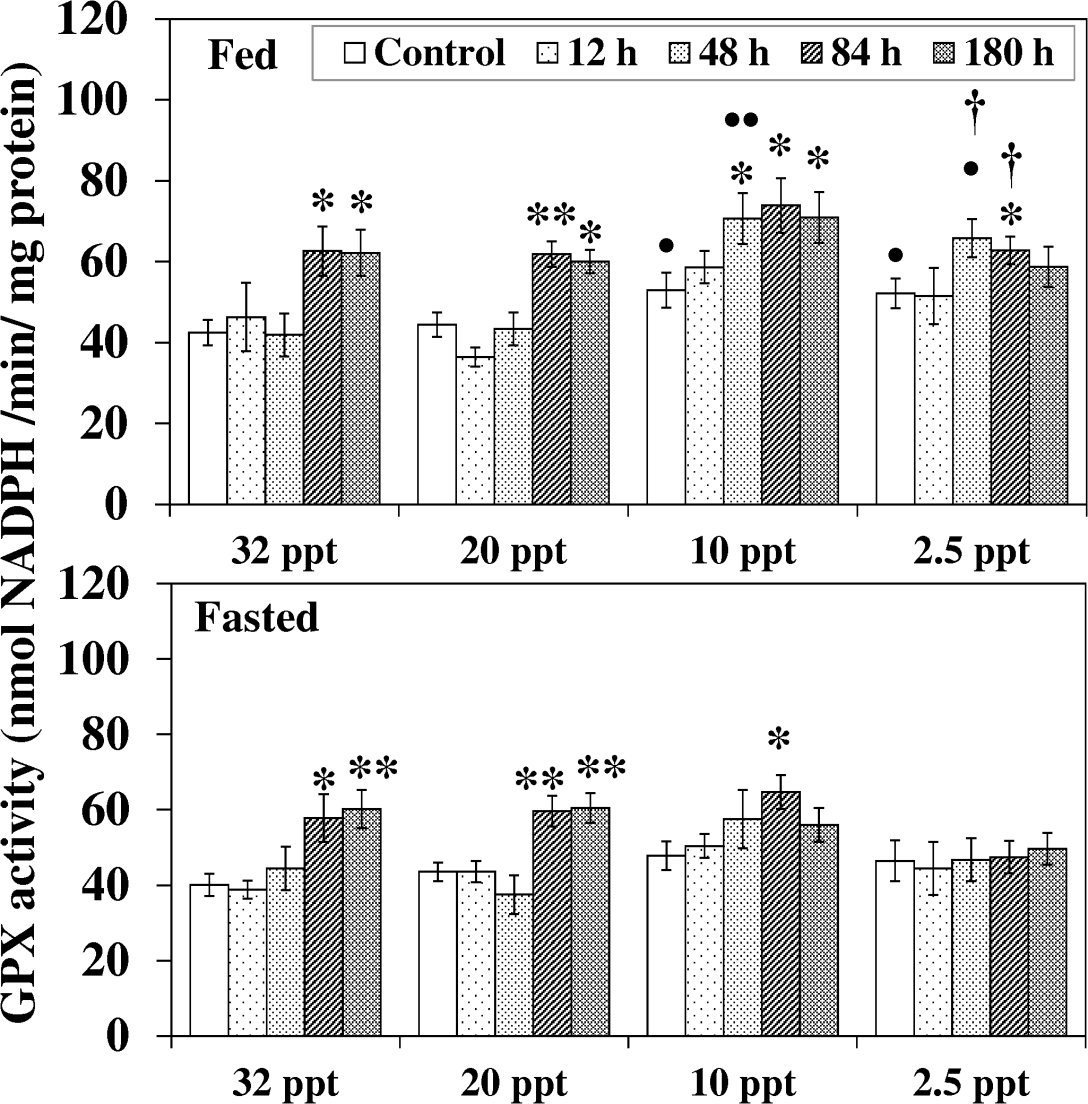
Glutathione peroxidise activity. Glutathione peroxidise (GPX) activity (nmol NADPH/min/mg protein) in liver of fed and fasted fish during acclimation to different salinities and exposure to HEA. Values are mean ± S.E. Asterisk (*) indicates a significant difference between the ammonia exposed fish and its respective control at each salinity (*P < 0.05; **P < 0.01), bullet (•) indicates a significant difference between experimental salinities (20 ppt -2.5 ppt) and the 32 ppt-acclimated fish at the same sampling period (^•^ *P* < 0.05; ^••^ *P* < 0.01), dagger (†) denotes the significant difference between fed fish and its respective fasted fish counterpart (^†^ *P* < 0.05).

Acclimation to lower salinity (10 ppt and 2.5 ppt) induced a considerable increment in GPX activity in fed fish relative to their 32 ppt control group (Fig 7). HEA exposure at 32 ppt and 20 ppt augmented (*P* < 0.05–0.01) the activity in both fed and fasted fish from 84 h onwards. At 10 ppt, such elevation (*P* < 0.05) in fed fish was seen from 48 h and continued till the end while a transient increment at 84 h in fasted fish was followed by a decline at 180 h HEA exposure. HEA exposure (during 84 h) at a hyposaline environment (2.5 ppt) only caused a rise in GPX activity in the fed fish, and this increment was higher (*P* < 0.05) compared to their parallel fasted fish group.

**Fig 8:**
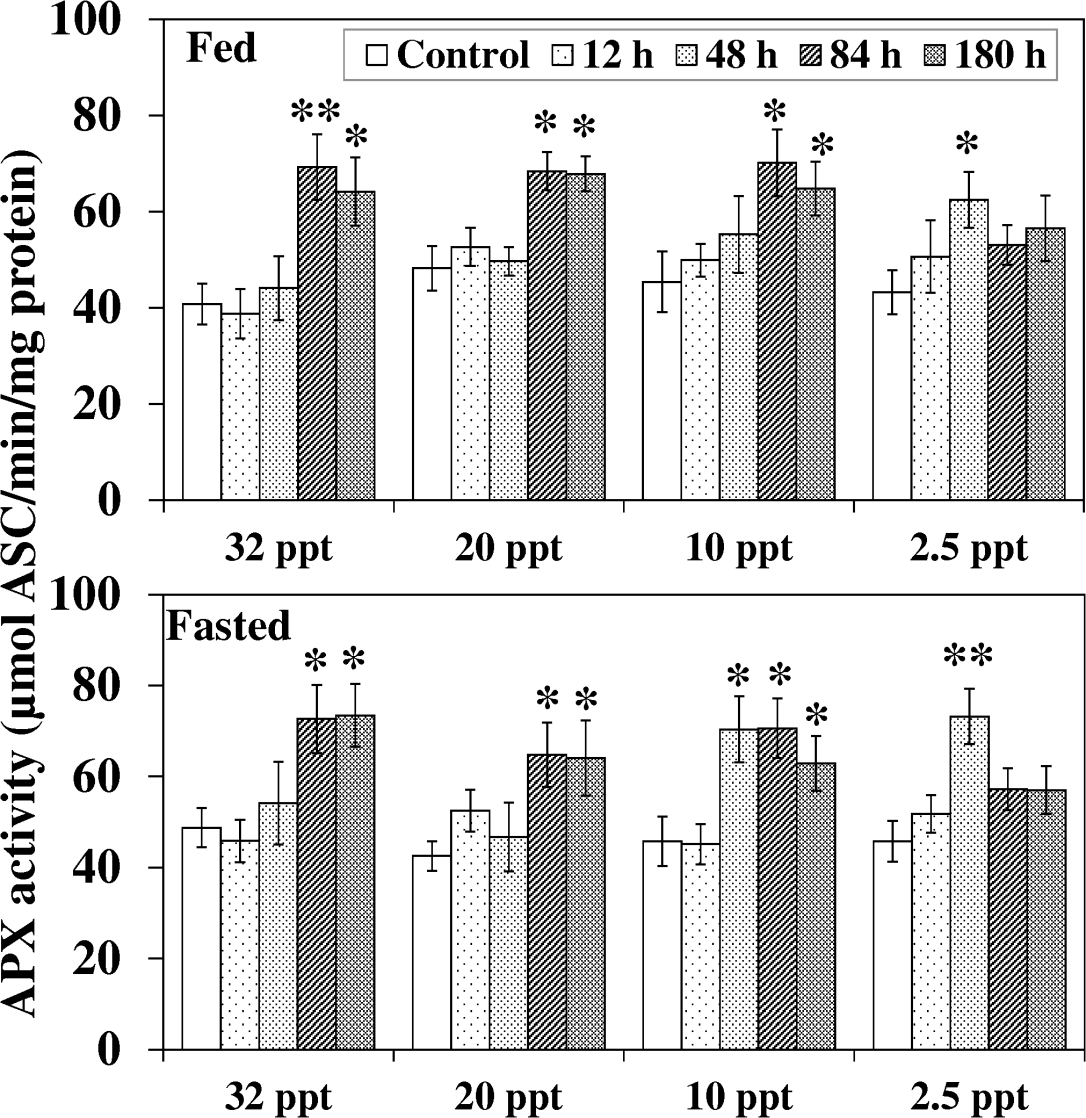
Ascorbate peroxidase acivity. Ascorbate peroxidase (APX) acivity (μmol ASC/min/mg protein) in liver of fed and fasted fish during acclimation to different salinities and exposure to HEA. Values are mean ± S.E. Asterisk (*) indicates a significant difference between the ammonia exposed fish and its respective control at each salinity (*P < 0.05; **P < 0.01).

Fed and fasted fish displayed an almost similar pattern for APX activity in response to ammonia exposure at 32 ppt and 20 ppt; a significant increment was noted at 84 h and 180 h exposure (Fig 8). At 10 ppt, increment in fed fish and fasted fish was observed after 84 h and 48 h respectively. At 2.5 ppt, the increment in fed and fasted fish was apparent (*P* < 0.05–0.01) at 48 h, which declined to control level afterwards.

#### Ascorbate- Glutathione cycle (GSH, ASC, their redox, GR and DHAR)

Acclimation to low salinity (10 ppt and 2.5 ppt) induced an increase in [GSH] in hepatic tissue of fed fish whereas fasted fish did not respond in function of salinity change (Table 1). An increase (*P* < 0.05) in the GSH/GSSH ratio in function of salinity reduction was observed for fed fish at 2.5 ppt (Table 1). Chronic HEA exposure (84 h-180 h) at 32 ppt-10 ppt increased (*P* < 0.05) the [GSH] in fed fish, and a comparable augmentation for fasted fish was seen at 32 ppt and 20 ppt although the value declined at the last exposure period (Table 1). HEA at 2.5 ppt induced a significant (transient) elevation at 48 h exposure for fed fish compared to the respective control, 32 ppt exposed fish and the corresponding 2.5 ppt exposed fasted fish. HEA exposure at 32 ppt and 20 ppt at 84 h-180 h raised the GSH/GSSH ratio in both fed and fasted fish (Table 1). At 10 ppt, following chronic (84 h-180 h) HEA exposure, the ratio increased significantly in fed fish compared to the control, the corresponding fasted fish, and 32 ppt exposed fed fish. Though a temporary increment (at 84 h) for HEA exposed fasted fish was observed at 10 ppt, no change was seen at 2.5 ppt. On the contrary, at this lowest salinity (2.5 ppt) GSH/GSSH ratio in fed control and HEA exposed fish was many folds higher (*P* < 0.05) than in the respective fasted fish.

**Table 1:** Glutathione (GSH), GSH/oxidized glutathione (GSSH) ratio, Ascorbate (ASC), ASC/Dehydroascorbate (DHA) ratio, GST (nmol GSH CDNB conjugates/min/mg protein) and DHAR (nmol ASC/min/mg protein) activity in the liver of European sea bass under the experimental conditions.

Treatment		GSH (nmol/g)		GSH/GSSH		ASC (nmol/g)		ASC/ DHA		GST		DHAR	
		Fed	Fasted	Fed	Fasted	Fed	Fasted	Fed	Fasted	Fed	Fasted	Fed	Fasted
32 ppt	Control	898 ± 32	865 ± 34	5.34 ± 0.45	5.76 ± 0.41	607 ± 17	573 ± 20	5.63 ± 0.36	5.42 ± 0.31	123 ± 6	128 ± 6	2.26 ± 0.21	2.43 ± 0.19
	12 h	872 ± 42	948 ± 46	5.78 ± 0.62	5.60 ± 0.55	571 ± 33	578 ± 36	5.23 ± 0.55	5.31 ± 0.50	125 ± 10	131 ± 7	2.21 ± 0.26	2.43 ± 0.25
	48 h	991 ± 53	973 ± 57	5.26 ± 0.59	5.88 ± 0.57	732 ± 29**	688 ± 31*	5.65 ± 0.59	6.78 ± 0.54*	136 ± 9	126 ± 11	2.67 ± 0.40	3.66 ± 0.33*
	84 h	1076 ± 63*	1174 ± 59**	6.19 ± 0.55	6.13 ± 0.60	702 ± 28*	649 ± 23*	7.21 ± 0.63*	7.75 ± 0.80**	128 ± 9	129 ± 6	3.50 ± 0.34*	3.57 ± 0.55
	180 h	1111 ± 73*	926 ± 60	6.75 ± 0.59*	7.45 ± 0.70*	712 ± 38*	647 ± 24*	6.90 ± 0.47*	7.45 ± 0.67**	131 ± 5	123 ± 6	3.29 ± 0.27*	3.40 ± 0.31*
20 ppt	Control	891 ± 36	917 ± 32	5.26 ± 0.41	5.32 ± 0.39	577 ± 19	585 ± 29	5.56 ± 0.36	5.41 ± 0.38	138 ± 6	140 ± 7	2.25 ± 0.18	2.68 ± 0.21
	12 h	950 ± 36	884 ± 68	5.08 ± 0.45	5.00 ± 0.42	580 ± 30	615 ± 31	5.67 ± 0.38	5.86 ± 0.66	133 ± 8	133 ± 5	2.83 ± 0.39	2.86 ± 0.31
	48 h	1102 ± 38*	988 ± 54	5.11 ± 0.39	5.89 ± 0.39	638 ± 22	676 ± 24*	5.7 ± 0.42	6.77 ± 0.59*	151 ± 6	153 ± 6	2.54 ± 0.37	3.52 ± 0.28*
	84 h	1157 ± 66*	1276 ± 89**	6.81 ± 0.44*	6.88 ± 0.63*	695 ± 32*	664 ± 31	7.35 ± 0.44**	7.39 ± 0.60**	143 ± 8	141 ± 17	3.87 ± 0.42*	4.31 ± 0.51*
	180 h	1066 ± 36*	1067 ± 62	6.95 ± 0.45*	7.44 ± 0.64*	658 ± 18*	674 ± 26*	7.06 ± 0.52*	7.24 ± 0.58*	148 ± 8	133 ± 5	3.19 ± 0.28*	3.78 ± 0.27*
10 ppt	Control	1058 ± 65**•**	966 ± 54	6.38 ± 0.59	5.78 ± 0.66	611 ± 29	610 ± 23	5.21 ± 0.51	5.31 ± 0.58	148 ± 8**•**	147 ± 7**•**	2.49 ± 0.21	2.62 ± 0.22
	12 h	1047 ± 56**•**	1032 ± 76	6.12 ± 0.67	5.99 ± 0.51	641 ± 34	647 ± 20	5.11 ± 0.57	5.67 ± 0.60	158 ± 7**•**	149 ± 13	2.55 ± 0.31	2.91 ± 0.40
	48 h	1073 ± 103	1193 ± 42**•**	6.08 ± 0.66	5.83 ± 0.67	644 ± 19	652 ± 27	6.79 ± 0.60	6.48 ± 0.64	157 ± 8	159 ± 8**•**	2.49 ± 0.52	3.59 ± 0.40
	84 h	1219 ± 48*	1092 ± 89	9.79 ± 0.84** ^••^ **†**	7.95 ± 0.71* **•**	715 ± 20**	717 ± 26*	6.43 ± 0.66	7.35 ± 0.62*	158 ± 7**•**	162 ± 8**•**	3.27 ± 0.44	4.02 ± 0.38*
	180 h	1208 ± 42*	1121 ± 57**•**	8.65 ± 0.67** **•†**	6.96 ± 0.70	695 ± 20*	692 ± 22**	7.19 ± 0.60*	7.17 ± 0.68*	155 ± 8**•**	151 ± 10**•**	3.95 ± 0.53*	3.95 ± 0.44*
2.5 ppt	Control	1000 ± 49**•**	943 ± 55	6.76 ± 0.59**•†**	5.08 ± 0.44	645 ± 31	612 ± 33	5.67 ± 0.55	5.66 ± 0.54	144 ± 8**•**	140 ± 6**•**	2.89 ± 0.30	2.78 ± 0.26
	12 h	1057 ± 68**•**	962 ± 48	6.89 ± 0.74**†**	5.14 ± 0.62	668 ± 26	595 ± 40	5.44 ± 0.47	6.12 ± 0.47	156 ± 7**•**	151 ± 8	2.55 ± 0.47	3.40 ± 0.42
	48 h	1181 ± 61* **•†**	996 ± 46	6.79 ± 0.56**†**	5.29 ± 0.52	709 ± 34	731 ± 21*	6.08 ± 0.55	5.89 ± 0.43	143 ± 5	161 ± 7**•**	3.04 ± 0.35	3.37 ± 0.51
	84 h	1079 ± 60	1076 ± 47	8.79 ± 0.68* **•†**	6.15 ± 0.54	733 ± 27*	680 ± 29	7.39 ± 0.67*	7.47 ± 0.78*	158 ± 9**•**	156 ± 8**•**	3.24 ± 0.34	4.19 ± 0.45*
	180 h	1066 ± 59	1024 ± 50	8.07 ± 0.66**†**	6.04 ± 0.66	711 ± 26	672 ± 40	6.73 ± 0.70	6.04 ± 0.60	151 ± 7**•**	149 ± 7**•**	3.81 ± 0.33	4.02 ± 0.34*

Values are mean ± S.E. Asterisk (*) indicates a significant difference between the ammonia exposed fish and its respective control at each salinity

*P < 0.05

**P < 0.01)

Bullet (•) indicates a significant difference between experimental salinities (20 ppt -2.5 ppt) and the 32 ppt-acclimated fish at the same sampling period

^•^ *P* < 0.05

^••^ *P* < 0.01

Dagger (†) denotes the significant difference between fed fish and its respective fasted fish counterpart

^†^ *P* < 0.05

**Fig 9:**
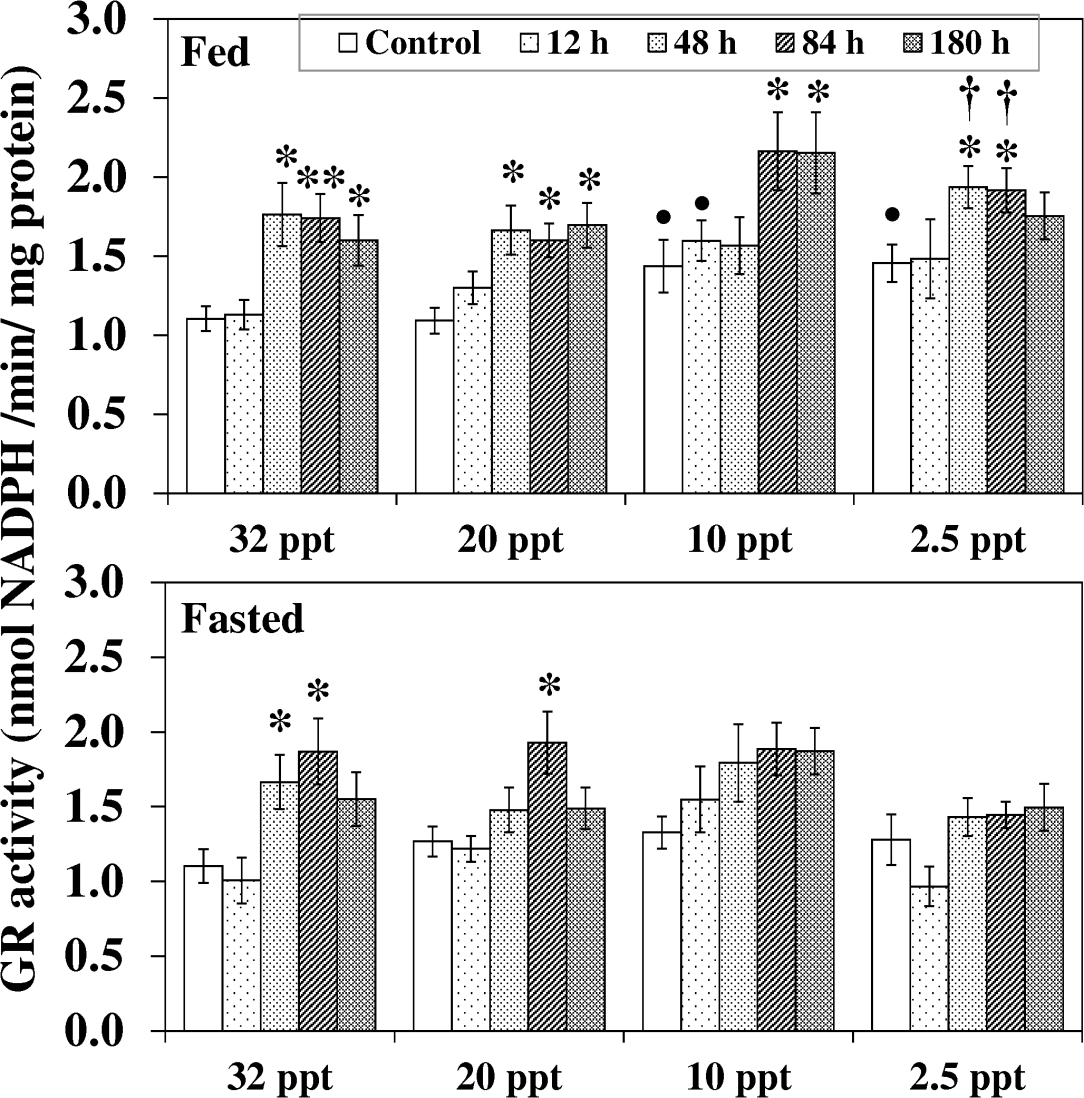
Glutathione reductase activity. Glutathione reductase (GR) activity (nmol NADPH/min/mg protein) in liver of fed and fasted fish during acclimation to different salinities and exposure to HEA. Values are mean ± S.E. Asterisk (*) indicates a significant difference between the ammonia exposed fish and its respective control at each salinity (*P < 0.05; **P < 0.01), bullet (•) indicates a significant difference between experimental salinities (20 ppt -2.5 ppt) and the 32 ppt-acclimated fish at the same sampling period (^•^ *P* < 0.05), dagger (†) denotes the significant difference between fed fish and its respective fasted fish counterpart (^†^ *P* < 0.05).

Similar to GPX, under control conditions, fed fish at 10 ppt and 2.5 ppt showed significantly higher GR activity compared to the respective 32 ppt control group (Fig 9). HEA exposure at 32 ppt and 20 ppt resulted in an increase in GR activity in both fed and fasted fish. In fed fish, a noteworthy increment was seen from 48 h onwards while in feed deprived individual a transient increment at 48 h-84 h was followed by a reduction to control level. Though no increment was observed for HEA exposed fasted fish acclimated to lower salinities (10 ppt—2.5 ppt), fed fish at these lower salinities showed a significant increment in GR activity. At 10 ppt, compared to the control, a significant increment of 52% and 50% respectively after 84 h and 180 h exposure was noted for fed fish. At 2.5 ppt, activity in fed fish following 48 h -84 h of HEA exposure intensified by 36% (*P* < 0.05) and 33% (*P* < 0.05) relative to the control, and these elevations were also extensively higher (*P* < 0.05) than the corresponding fasted exposed group.

No individual effect of salinity was observed for [ASC] and ASC/DHA ratio (Table 1). Exposure of sea bass to HEA induced an increase of [ASC] in both feeding treatments. At 32 ppt, the increment (*P* < 0.05–0.01) was notable from 48 h onwards while at 20 ppt and 10 ppt the significant elevation was documented after 84 h. However, at the lowest salinity, both feeding treatments manifested a decline towards the last HEA exposure periods. The ratio of ASC to DHA revealed roughly the same pattern as seen for [ASC].

DHAR activity in fed fish at 32 ppt and 20 ppt elevated significantly in response to 84 h-180 h HEA exposure, the time response was shorter in fasted fish and was significant from 48 h onwards (Table 1). Similarly, at 10 ppt the activity in fasted fish was enhanced (*P* < 0.05) from 84 h onwards while a delayed response (at 180 h) was apparent in fed fish. Such increment (*P* < 0.05) in response to HEA remained elevated in fasted fish even at 2.5 ppt acclimation; however no stimulation was seen for fed fish.

#### Detoxification enzyme

A significant effect of low salinity acclimation was seen on GST activity (Table 1). Following acclimation to 10 ppt and 2.5 ppt, GST activity increased significantly in both fed and fasted fish compared to the parallel control and HEA exposed group acclimated at normal seawater salinity. However, no effect of HEA was noted for GST activity in both fed and fasted fish at different salinity regimes.

### Principal component analysis (PCA) and Correlation analysis

**Fig 10:**
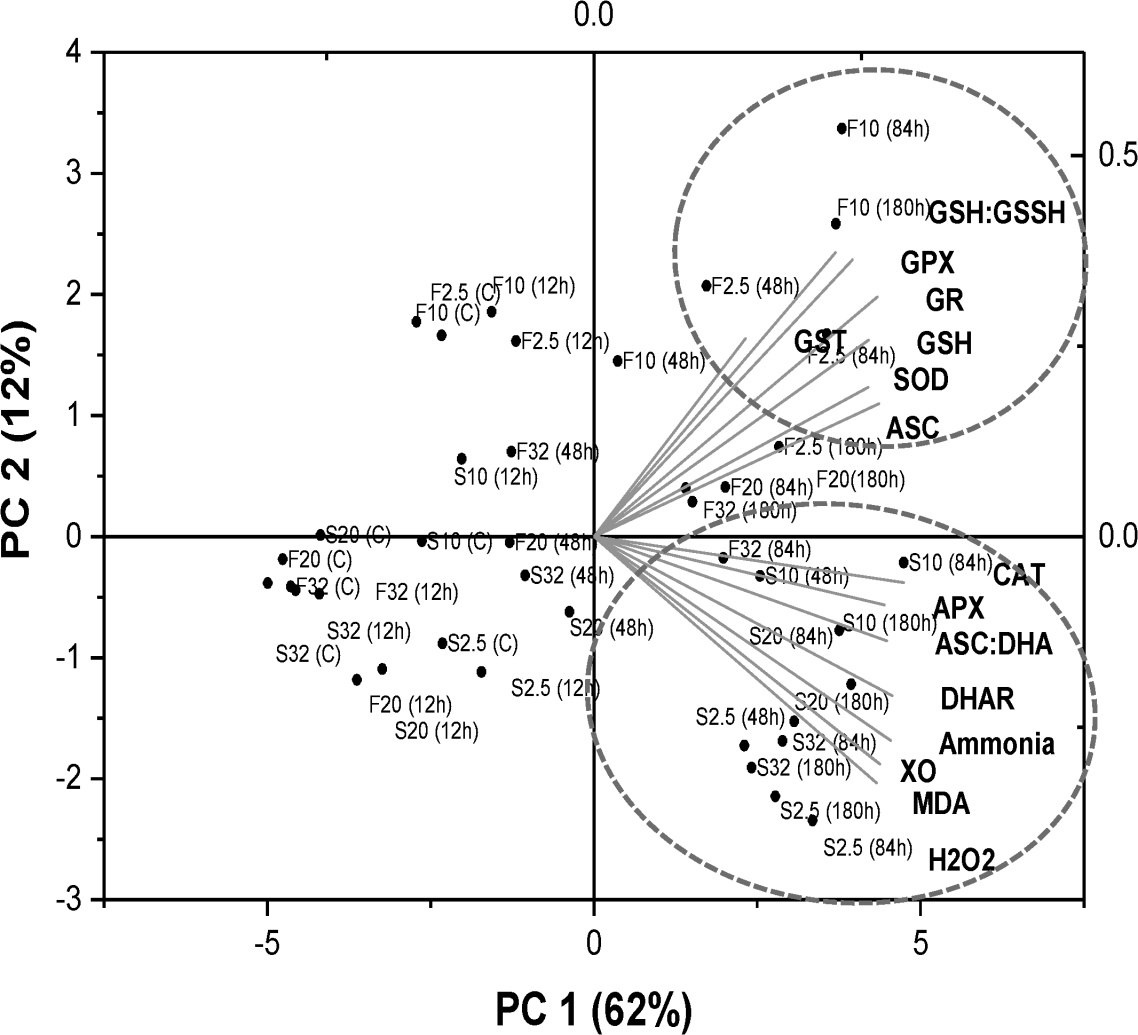
Principal Component Analysis. Principal Component Analysis (PCA) representing the contribution of biochemical parameters for fed (F) and starved (S) fish. The variable coordination is presented by the complementary cases analysis showing distribution of salinity acclimation groups (32, 20, 10, and 2.5 ppt) and HEA exposure (C, 12 h, 48 h, 84 h, 180 h) in the (PC 1 ×PC 2) coordination plane.

The PCA biplot of overall data depicts a clear separation of experimental groups, mainly along the first two components (PC 1 and PC 2), together explaining 74% of data variability (Fig 10). These two components showed a clear separation among fed and fasted fish which was prominent during the combined effect of chronic HEA exposure (48 h-180 h) and reduced seawater salinities (20 ppt-2.5 ppt). The prevailing PC 1 component (62% of the data variance) clustered oxidative stress indices (H_2_O_2_, MDA, XO), ammonia accumulation, APX, DHAR and CAT activity with HEA exposed fasted fish held at low salinities. PC 2 (12% of the data variance) clustered low salinity acclimated HEA exposed fed fish with SOD, ASC content and components of glutathione dependent antioxidant system.

Pearson correlation among the various variables in fed and fasted fish under experimental condition is represented in Table 2. The interaction between all the three experimental factors (salinity stress, ammonia and nutritional status) for all the different parameters investigated in the present study is illustrated in S1 Table.

**Table 2:** Correlations among various variables investigated in the liver of fed and fasted European sea bass during individual and combined effect of salinity and ammonia.

Experimental conditions		Amm. content	H_2_O_2_	MDA	XO	SOD	CAT	GPX	GR	GSH	GST	APX	ASC
**Fed**													
H_2_O_2_	Sal.	-0.075											
	Amm	0.302											
	Sal x Amm	0.161*											
MDA	Sal.	0.184	0.049										
	Amm	0.214	0.229										
	Sal x Amm	0.160*	0.214*										
XO	Sal.	0.234	-0.004	0.193									
	Amm	0.094	0.034	0.121									
	Sal x Amm	0.253*	0.029	0.103									
SOD	Sal.	-0.214	0.058	-0.162	0.006								
	Amm	0.102	0.282	-0.004	0.163								
	Sal x Amm	0.163*	0.210*	0.058	0.086								
CAT	Sal.	0.123	0.096	0.064	-0.108	0.137							
	Amm	0.165	0.003	0.267	0.275	0.085							
	Sal x Amm	0.201**	0.056	0.169*	0.154*	0.333**							
GPX	Sal.	0.004	0.122	-0.027	0.167	0.386**	0.103						
	Amm	0.004	0.082	0.146	0.02	0.106	0.284						
	Sal x Amm	0.073	0.134	0.076	0.166*	0.301**	0.191*						
GR	Sal.	0.173	-0.281*	0.182	-0.042	0.093	0.226	0.034					
	Amm	0.153	0.299	0.248	0.342*	0.294	0.354*	0.115					
	Sal x Amm	0.186*	0.150	0.151	0.198**	0.266**	0.291**	0.239**					
GSH	Sal.	-0.102	-0.215	-0.131	-0.202	0.215	-0.002	0.112	0.311*				
	Amm	0.095	0.056	0.101	0.146	0.266	0.152	0.173	0.146				
	Sal x Amm	0.025	-0.007	0.165*	0.095	0.264**	0.193*	0.245**	0.320**				
GST	Sal.	-0.112	-0.082	0.033	0.098	0.124	0.07	0.206	0.025	0.041			
	Amm	0.178	-0.226	-0.091	-0.018	0.068	-0.222	0.111	-0.028	0.176			
	Sal x Amm	0.091	0.014	0.055	0.1	0.153	0.138	0.274**	0.108	0.166*			
APX	Sal.	0.154	0.193	0.281*	0.081	0.22	0.187	0.313*	0.071	-0.166	-0.241		
	Amm	0.179	0.284	0.367*	0.282	0.303*	0.206	0.222	0.466**	0.152	-0.063		
	Sal x Amm	0.148	0.199*	0.272**	0.236**	0.266**	0.201**	0.258**	0.213**	0.248**	-0.006		
ASC	Sal.	0.199	0.241	0.189	0.252	0.391**	0.138	0.361**	0.257	0.003	0.013	0.236	
	Amm	-0.183	0.014	0.152	0.439**	0.453**	0.208	0.173	0.619**	0.336*	0.001	0.376**	
	Sal x Amm	0.206**	0.173*	0.270**	0.247**	0.419**	0.223**	0.236**	0.334**	0.234**	0.005	0.268**	
DHAR	Sal.	-0.024	-0.018	0.034	0.067	0.303*	0.379*	0.297*	0.366*	0.220	0.016	0.374**	0.221
	Amm	0.005	0.092	0.247	0.21	0.16	0.238	0.330*	0.341*	0.234	0.165	0.353*	0.452**
	Sal x Amm	0.157*	0.076	0.097	0.125	0.324**	0.246*	0.192*	0.227*	0.156	0.092	0.300**	0.336**
**Fasted**													
H_2_O_2_	Sal.	0.189											
	Amm	0.436*											
	Sal x Amm	0.310*											
MDA	Sal.	0.048	0.352*										
	Amm	0.259	0.414*										
	Sal x Amm	0.274*	0.353*										
XO	Sal.	-0.006	0.138	-0.191									
	Amm	0.334*	0.158	0.338*									
	Sal x Amm	0.243**	0.267**	0.238**									
SOD	Sal.	-0.009	-0.111	0.103	-0.039								
	Amm	0.264	0.135	.455**	0.057								
	Sal x Amm	0.235**	0.153*	0.341**	0.206**								
CAT	Sal.	0.170	0.232	0.035	0.239	0.160							
	Amm	0.297*	0.410**	0.149	0.524**	0.161							
	Sal x Amm	0.341**	0.317**	0.276**	0.435**	0.321**							
GPX	Sal.	-0.094	-0.111	-0.006	0.158	0.102	0.016						
	Amm	0.375*	0.264	0.293	0.131	0.358*	0.306*						
	Sal x Amm	0.155*	0.151	0.149	0.260**	0.215**	0.156*						
GR	Sal.	0.006	-0.033	-0.007	-0.101	0.063	0.039	0.197					
	Amm	0.323*	0.275	0.149	0.236	0.296	0.338*	0.330*					
	Sal x Amm	0.272**	0.204**	0.149	0.186*	0.127	0.271**	.206**					
GSH	Sal.	0.022	0.131	0.094	0.077	0.132	0.353**	-0.042	0.235				
	Amm	0.271	0.408**	0.211	0.282	-0.004	0.295	0.022	0.216				
	Sal x Amm	0.265**	0.257**	0.270**	0.293**	0.113	0.244**	0.210**	0.347**				
GST	Sal.	0.291*	0.259	0.155	0.196	0.025	0.322*	0.124	0.033	0.294*			
	Amm	0.110	-0.09	-0.085	-0.02	0.040	0.144	-0.007	-0.137	0.186			
	Sal x Amm	0.272**	0.145	0.119	0.187*	0.099	0.205**	0.126	0.079	.235**			
APX	Sal.	0.009	-0.007	0.316*	-0.238	-0.053	-0.135	-0.097	0.033	-0.102	-0.056		
	Amm	0.189	0.272	0.423**	0.13	0.16	-0.058	0.279	0.217	0.138	-0.196		
	Sal x Amm	0.261**	0.283**	.310**	0.113	0.112	0.170*	0.204**	0.275**	0.172*	-0.012		
ASC	Sal.	0.194	0.164	0.173	-0.162	0.081	0.141	0.079	0.165	-0.168	0.279**	0.024	
	Amm	0.241	0.176	0.133	-0.047	0.159	0.203	0.438**	0.380*	0.134	0.09	-0.05	
	Sal x Amm	0.274**	0.275**	0.272**	0.180*	0.202**	0.319**	0.180*	0.181*	0.09	0.200**	0.134	
DHAR	Sal.	-0.003	-0.037	0.034	0.182	-0.086	-0.08	0.091	0.199	-0.13	-0.033	0.041	-0.143
	Amm	0.26	0.592***	0.332*	0.395*	0.159	0.385*	0.200	0.201	0.275	-0.123	0.156	0.135
	Sal x Amm	0.247**	0.354**	0.279**	0.288**	0.153	0.230**	0.134	0.221**	0.132	-0.034	0.241**	0.11

The listed values are the correlation coefficient (r).

*Correlation is significant at 0.05 level (2-tailed).

**Correlation is significant at 0.01 level (2-tailed).

## Discussion

### Starvation exacerbates oxidative stress induced by salinity, ammonia and their combination

We examined the level of H_2_O_2_ and MDA production along with the activity of XO, as biomarkers of oxidative stress. The isolated effect of salinity challenge demonstrates that oxidative stress was induced when sea bass were exposed to reduced seawater salinities and that it was more prominent during feed deprivation. Unlike in fed fish, the production of H_2_O_2_ in the liver of fasted fish elevated in response to salinity stress (10 ppt-2.5 ppt). MDA, one of the major products of lipid peroxidation increased in response to salinity reduction, and similar to H_2_O_2_ the response was elevated only in fasted fish. This suggests that generation of ROS was induced as a result of the synergistic effect of hyposaline environment and starvation, eventually propagating a pro-oxidant condition. Likewise, MDA level was reported to increase in hepatic tissue of gilthead seabream (*Sparus aurata*) during partial or total food deprivation [32]. Salinity stress was also reported to considerably enhance the production of lipid peroxidation in sturgeons (*Acipenser naccarii*) [54], however interactive effect of salinity and feeding on oxidative stress level has not been studied before.

High ammonia in water is associated with oxidative stress in fish [23–26]. Increases in lipid peroxidation in response to ammonia exposure has been documented in many teleosts [24–26]. In the present study, exposure to 20 mg/L ammonia at normal seawater (32 ppt) as well as at reduced salinities (20 ppt-2.5 ppt) led to a substantial increase in ammonia accumulation in the liver of both fed and fasted fish, and was accompanied by a differential and time-dependent response of oxidative stress markers. The response of H_2_O_2_ and MDA content in both feeding groups were positively (*P* < 0.05–0.001) correlated with ammonia accumulation in the hepatic cells, meaning that elevated internal ammonia levels in combination with salinity reduction may be involved in the signaling mechanism for stimulating free radical production. In addition, elevated H_2_O_2_ and MDA levels in fasted fish failed to re-establish to control levels in contrast to the fed fish. It implies that food availability is a crucial factor restraining the oxidative status of fish during the stressful condition, which was also clear from the PC clusters analysis. Similar with our results, increases in lipid peroxidation levels under conditions of food deprivation was also reported in the liver of rainbow trout (*Oncorhynchus mykiss*) [38,55], gilthead seabream [32], common dentex (*Dentex dentex*) [56] and Adriatic sturgeon (*Acipenser naccarii*) [38]. Till date, no information is available concerning the mutual effect of food deprivation, salinity reduction and ammonia threat on the oxidative status in fish.

Furthermore, the precise mechanisms by which ammonia and salinity stress induce free radical production and oxidative stress are poorly understood in fish. In response to ammonia intoxication, the over-activation of *N*-methyl-D-aspartate type glutamate (NMDA) receptors in nervous tissue is proposed to incite excess production of ROS [57]. However, hepatic tissue lacks NMDA receptors, suggesting another route by which HEA can cause oxidative damage in liver [26]. Nevertheless, ROS are also formed by XO which utilize molecular oxygen as the electron acceptor and liberates considerable amounts of superoxide anion (O_2_^• −^) and H_2_O_2_ [58,59]. A positive correlation (*P* < 0.01–0.001) between ammonia build-up and XO activity emphasize that stimulation of XO could be an alternative ROS production pathway mediated by ammonia toxicity. Our result is also consistent with earlier findings in Nile tilapia (*Oreochromis niloticus*), common carp (*Cyprinus carpio*), goldfish (*Carassius auratus*) and rainbow trout favoring the activation of XO as a potential factor stimulating ROS production under ammonia exposure [26,34]. Furthermore, in fasted fish the highly elevated XO activity at lower salinities (10 ppt-2.5 ppt) and during HEA exposure (at 10 ppt and 2.5 ppt) is presumably responsible for persistent occurrence of significant higher H_2_O_2_ and MDA content compared to the fed fish. This effect in fasted fish was also revealed by a positive correlation of XO with H_2_O_2_ (r = 0.267, *P* < 0.01) and MDA content (r = 0.238, *P* < 0.01). Oxidative stress induced by a low salinity might be associated with an increase in oxygen consumption owing to osmo-regulation. However, we do not have the data of oxygen consumption rate to support this premise. In addition, fish gills serve as the dynamic respiratory and ion-osmo regulatory organ, and are prone to the oxidative injury particularly during the osmotic stress. Consequently, future studies on oxidative status in branchial tissue are advised to link between the free radical generation and the fluctuation in the ambient seawater salinity.

Our findings further suggest that the oxidative damage induced by low salinity and HEA, isolated or combined, were exacerbated during feed withdrawal, and feeding could efficiently eliminate ROS including H_2_O_2_, restricting the accretion of terminal products of lipid per-oxidation (e.g. MDA). The alleviated oxidative stress level in fed fish might be due to higher levels of antioxidants as compared to fasted fish under unfavorable conditions as discussed below.

### Feeding ameliorates the antioxidant defensive system

#### ROS scavenging enzymes

Levels of ROS in the cells are controlled by the concerted action of various ROS scavenging enzymes. For instance, SOD catalyzes the conversion of O_2_^• −^ to H_2_O and H_2_O_2_, and the later is further degraded into H_2_O and O_2_ by CAT, APX, GPX and multiple different peroxidases [33]. SOD activity was elevated when the fish were subjected to low salinity conditions. Similar findings have been reported in juvenile silver pomfrets (*Pampus argenteus*) [12] and black rockfish (*Sebastes schlegeli*) [60] during low salinity challenge. Nevertheless, the consequence of increased SOD activity would result in more production of H_2_O_2_; the elevated activity of two H_2_O_2_-scavenging enzymes CAT and GPX in the fed fish in response to salinity stress might explain the restoration of H_2_O_2_ content to basal level. However, GPX activity did not respond in fasted fish, highlighting their limited ability (relative to the fed fish) in scavenging H_2_O_2_. In previous studies, starvation had either no effect on hepatic GPX activity in European sea bass [61] or significantly declined GPX activity in rainbow trout and Adriatic sturgeon [38]. It was also reported that food deprivation hinders the antioxidant defences for effective scavenging of the generated ROS in the liver of brown trout (*Salmo trutta*), leading to the appearance of lipid peroxidation [62].

In response to ammonia exposure at normal seawater salinity (32 ppt) and at reduced salinities (20, 10 and 2.5 ppt), an increment of SOD activity suggests that sea bass utilizes SOD as an initial defensive strategy against ammonia poisoning. Interestingly, the delayed response of SOD in fasted fish did not correspond with persistent increment in H_2_O_2_ level, suggesting an alternative route inducing H_2_O_2_ production. We found that H_2_O_2_ production followed a parallel increment of XO activity (r = 0.267, *P* < 0.01) which is also substantiated by a recent study confirming H_2_O_2_ as the dominant (70–95%) reactive product catalyzed by XO [34]. Thus our results signify that during ammonia and salinity threat, H_2_O_2_ production in liver particularly during feed limitation, is possibly regulated by XO. Furthermore, an elevated rate of GPX and CAT activity in fed fish in response to HEA at 20 ppt and 10 ppt might partially justify the recovery of H_2_O_2_ to the control level. However, the modest increment of GPX in fasted fish highlights their poor ability, relative to fed fish, in scavenging H_2_O_2_ resulting in a consistent elevated H_2_O_2_ level. Surprisingly, feed deprived sea bass responding to HEA at experimental salinities showed enhanced CAT activity relative to the fed fish. It is appealing to speculate that the improved responsiveness of CAT in fasted fish may be compensatory to the weak response of SOD and GPX activities. Therefore, CAT appears to have a more important role than GPX for cellular defence against H_2_O_2_. It is well documented that for the decomposition of H_2_O_2_ to H_2_O, CAT shares the antioxidant function with GPX; therefore, these two enzymes might complement or contend with each other in the H_2_O_2_ catabolism [12]. At low cellular H_2_O_2_ levels, organic peroxides are preferred by GPX, but during elevated H_2_O_2_, they are catalyzed by CAT [63]. In the present study, fasted fish had a high level of H_2_O_2_, favouring the CAT dependent pathway as the most preferred antioxidative mechanism during starvation. It is also seen in the PC1 clustering, emphasizing that CAT activity is primarily related to feed deprivation. The present finding is, in part, supported by the works of Aceto et al. [64], Wang et al. [65] and Fei et al. [66] confirming that CAT is relatively more prioritized than GPX for per-oxidation detoxification under low salinity stress.

In addition to CAT and GPX, APX is another enzyme responsible for the scavenging of cellular H_2_O_2_. The present study suggests an obscure role of APX as an antioxidant sentinel under salinity stress. The activation of hepatic APX in both feeding treatments signifies its potential role in controlling H_2_O_2_ particularly during ammonia threat. This also corroborates with our previous experiment that showed an elevated rate of APX activity in the liver of cyprinids in response to ammonia toxicity [26].

In brief, the kinetics of ROS scavenging enzymes in both feeding treatments revealed a similar pattern following individual effects of HEA (at 32 ppt). During the salinity stress alone and in the combined effect of HEA with salinities at 20 ppt and 10 ppt, the dynamics of these enzymes reflect advanced capability among fed fish over feed deprived fish to restrain H_2_O_2_ overproduction and thus to limit the cell damage. This might corroborates with the reduction of energy store (requisite for the efficient functioning of ROS scavenging enzymes) in hepatic tissue of fasted fish, witnessed from our previous study on European sea bass using the same experimental design [15].

#### GSH- dependent metabolic pathways and cellular detoxification

GSH acts as an effective antioxidant molecule besides serving as a substrate for GPX to neutralize H_2_O_2_ produced by SOD. Thus, cellular GSH store represents the competency of living organism to resist the oxidative damage. Under hypo-osmotic conditions (10 ppt -2.5 ppt), fed fish were characterized by augmented intracellular GSH pool. Similarly, ammonia exposure alone and in combination with salinity stress (except at 2.5 ppt) resulted in consistent increment in GSH content among fed fish, connoting an advantage for the fed fish to resist oxidative stress. GPX catalyses the reduction of H_2_O_2_ and a variety of lipid peroxides by using GSH which is further oxidized to GSSH. The cellular GSSH load is also an indicative of oxidative stress level. In general, GSSH is reduced to GSH via GR in a NADPH dependent reaction, and NADPH is generated by multiple redox enzymatic reactions that are markedly synchronized with nutrient supply [56,67–69]. As such, lower availability of NADPH as a consequence of nutrient limitation would curtail GR activity. This was reflected by an impaired GR activity in feed deprived fish accompanied by a decline in recycling rate of GSH regeneration from GSSG, signifying an incompetent GSH-recycling system in fasted fish under stressful experimental conditions. Similarly, depletion of endogenous GSH pool in fish has been reported to occur under a situation of food deprivation [29,70–72]. Pascual et al. [32] documented an elevated level of oxidized glutathione in partial or total food deprived gilthead seabream. In addition, the biological availability of sulphur amino acid determines the concentration of glutathione and glutathione-dependent enzymes [73,74]. The nutritional deficiency that fish were confronted to, could have restricted the availability of sulphur amino acids. This might detain the production of GSH and functional activities of GPX and GR in the fasted fish. Conversely, under different experimental conditions, fed fish retain a high GSH store and GSH/GSSG ratio probably signifying an efficient (relative to fasted fish) removal of GSSH from the cellular system. The parallel augmentation of GR with GPX activity in fed fish highlights a proficient renewal of GSH, suggesting a highly effective GSH-based antioxidant system than the fasted fish in mitigating the salinity and ammonia mediated oxidative damage.

GST promotes important cellular detoxification of xenobiotics or reactive molecules by forming conjugates with GSH, thus inhibiting the formation of lipid peroxides, hydroperoxide and their derivatives [9]. In this study, GST activity was mainly activated in response to hypo-osmotic stress irrespective of feeding treatment. The lipid compounds in hepatic tissue of sea bass seems to have a high tendency of being per-oxidized under the low salinity stress as evident by an increment in MDA content, thus the enhanced GST activity is possibly a counter response to minimize the lipid per oxidation. A similar increment of GST activity was also noted in juvenile silver pomfrets during osmotic stress [12]. Similar to earlier study in freshwater teleosts [26], the activity of GST remained unaltered during ammonia exposure emphasizing its insignificant role in ammonia detoxification.

#### ASC- redox cycle

ASC is a potent antioxidant which scavenges reactive radicals. Therefore, ASC and ASC/DHA ratio is recommended as the indices of the defensive response [75,76]. ASC content and ASC/DHA ratio remained unaltered in response to salinity challenge suggesting its insignificant role in restraining the adverse consequences of salinities fluctuations. Nevertheless, to our best knowledge there is no information available on the effect of salinity challenge on ASC content. An augmented ASC pool and ASC/DHA in both fed and fasted fish against HEA exposure at different salinities might be an adaptive approach to restrict the oxidative damage. An increased ASC content in hepatic tissue of goldfish and common carp was also reported in response to sublethal dose of ammonia [26]. ASC also serve as a substrate for APX in scavenging H_2_O_2_, consequently, a high activity of APX (discussed earlier) is expected to deplete cellular ASC content. In this study, the rise of APX activity was not coupled by a parallel decline of [ASC], suggesting an efficient supply of ASC from other routes. In the ascorbate-glutathione pathway, dehydroascorbate reductase (DHAR) regenerates ASC by reducing DHA and oxidizing GSH to GSSH. The parallel increment of DHAR activity facilitates an efficient supply of ASC at the cost of GSH which might hamper GSH content. However, DHAR activity was not correlated to the GSH content (Table 2), signifying ascorbate regeneration, at least in sea bass, is independent of GSH.

Overall, it is clear that feed limitation is the key environmental cue which can further exacerbate the oxidative injury incite by the hypo-osmotic stress and ammonia toxicity. It can be advised that whenever the threat of salinity reduction and ammonia pollution is anticipated, feeding should be continued for a better farm management. Additionally, our study provides a cautionary note as to the importance of considering feeding while formulating the frameworks for the regulation of certain interacting environmental factors influencing the sustainability of natural as well as mariculture systems.

## Conclusion

The present study shows that the individual and combined effect of salinity stress and high environmental ammonia can incite pro-oxidant conditions, manifested by the occurrence of oxidative stress and antioxidant compensatory responses, which were modulated differentially in fed and fasted fish. In response to osmotic challenge as a single factor, fed fish were able to avoid oxidative stress evident by the retention of H_2_O_2_ and MDA content at the control level (32 ppt), coincided by the persistent, simultaneous augmentations of both enzymatic (SOD, CAT, GPX, GR and GST activity) and non-enzymatic (GSH content) antioxidants. Unlike in fed fish, the hypo-osmotic stress (10 ppt-2.5 ppt) is detrimental for fasted fish and thus might limit their ability to cope with the reduced seawater salinities. HEA itself (at 32 ppt) also induced H_2_O_2_ and MDA production. In fed fish, the significant increment in the antioxidant system was accompanied by a decline in the oxidative stress towards the last exposure period (180 h) whereas feed deprived fish failed to re-store at the basal level. Besides SOD, CAT and APX activation in both feeding groups during HEA exposure, the components of GSH cycle was predominantly activated in fed fish highlighting their additional ability to deal with ammonia induced lipid peroxidation. Furthermore, the conjoint salinity and HEA effect induced the changes in the magnitude of oxidative stress and the status of antioxidant defence differently compared to their individual exposure. Moreover, different patterns of induction were also revealed among fed and fasted fish. Following the HEA at 20 ppt and 10 ppt salinities, SOD and CAT activity increased in both feeding treatments. Unlike fed fish, fasted fish did not seem to effectively implement glutathione redox pathways but relied substantially on the CAT activity to counteract the ROS generation. ASC level augmented in both feeding treatments which was coupled with subsequent increment of APX and DHAR activity. Overall, it is apparent that the antioxidative defence system during combined effect of salinity (20 ppt-10 ppt) and HEA was effectively activated in fed sea bass. As such feeding can efficiently mitigate the oxidative cellular damage during the isolated and simultaneous effect of salinity stress and ammonia, while nutrient deprivation restrain the fish’s ability to counteract the ROS generation evident by persistent elevated levels of H_2_O_2_ and MDA in fasted fish. Nevertheless, similar to fasted fish, synergistic response of ammonia under conditions of hyposaline water (2.5 ppt) was deleterious even for the fed fish, and although a temporary activation of the antioxidant system was noted, it was not competent enough to effectively scavenge ROS production. Our study recommends that interactions between potential confounding environmental cues should be taken into account while assessing the ecological performance of aquatic animals.

## Supporting Information

S1 Table The effects of salinity, ammonia exposure and feeding status and their interactions on oxidative stress and anti-oxidant defence parameters in European sea bass. (DOC)
