# Supplementary material for: Nutritional Status as the Key Modulator of Antioxidant Responses Induced by High Environmental Ammonia and Salinity Stress in European Sea Bass (Dicentrarchus labrax)
Source: PLoS One. 2015 Aug 4;10(8):e0135091. doi: 10.1371/journal.pone.0135091 (PMC4524602; doi:10.1371/journal.pone.0135091)
Supplement: S1 Table — (DOC) [file pone.0135091.s001.doc]

**Table S1**

The effects of salinity, ammonia exposure and feeding status and their interactions on oxidative stress and anti-oxidant defence parameters in European sea bass

| Treatment | **Salinity** | | **Ammonia** | | **Feeding** | | **Salinity x Ammonia** | | **Salinity x Feed** | | **Ammonia x Feed** | | **Salinity x Ammonia x Feed** | |
| --- | --- | --- | --- | --- | --- | --- | --- | --- | --- | --- | --- | --- | --- | --- |
|  | *F* value | *P* value | *F* value | *P* value | *F* value | *P* value | *F* value | *P* value | *F* value | *P* value | *F* value | *P* value | *F* value | *P* value |
| Ammonia content | 1.46 | 0.226 | 23.25 | 0.000 | 8.66 | 0.003 | 0.511 | 0.907 | 2.00 | 0.113 | 1.05 | 0.378 | 0.246 | 0.996 |
| H_2_O_2_ | 2.82 | 0.039 | 26.49 | 0.000 | 14.92 | 0.000 | 0.48 | 0.921 | 0.52 | 0.665 | 3.16 | 0.014 | 0.93 | 0.510 |
| MDA | 3.39 | 0.018 | 27.47 | 0.000 | 25.57 | 0.000 | 1.27 | 0.235 | 0.42 | 0.734 | 4.85 | 0.001 | 0.58 | 0.858 |
| XO | 1.16 | 0.325 | 19.64 | 0.000 | 12.78 | 0.000 | 0.15 | 1.000 | 3.66 | 0.013 | 1.92 | 0.107 | 0.11 | 1.000 |
| SOD | 10.63 | 0.000 | 26.99 | 0.000 | 8.01 | 0.005 | 0.45 | 0.938 | 3.42 | 0.017 | 1.65 | 1.160 | 0.50 | 0.912 |
| CAT | 14.67 | 0.000 | 25.00 | 0.000 | 5.26 | 0.022 | 0.17 | 0.999 | 1.24 | 0.293 | 1.51 | 0.197 | 0.40 | 0.960 |
| GPX | 10.45 | 0.000 | 18.49 | 0.000 | 15.00 | 0.000 | 1.95 | 0.028 | 2.94 | 0.033 | 0.51 | 0.726 | 0.42 | 0.954 |
| GSH | 6.58 | 0.000 | 18.08 | 0.000 | 3.31 | 0.070 | 1.08 | 0.369 | 0.76 | 0.514 | 0.69 | 0.596 | 1.28 | 0.224 |
| GR | 6.24 | 0.000 | 22.78 | 0.000 | 6.10 | 0.014 | 0.95 | 0.497 | 3.03 | 0.029 | 0.52 | 0.717 | 0.68 | 0.770 |
| GST | 19.39 | 0.000 | 2.10 | 0.081 | 0.03 | 0.853 | 0.60 | 0.841 | 0.040 | 0.989 | 0.39 | 0.813 | 0.42 | 0.953 |
| APX | 0.48 | 0.694 | 22.83 | 0.000 | 1.49 | 0.000 | 2.36 | 0.006 | 1.30 | 0.274 | 0.40 | 0.802 | 0.22 | 0.997 |
| ASC | 3.66 | 0.013 | 21.34 | 0.000 | 2.45 | 0.118 | 9.67 | 0.480 | 1.99 | 0.115 | 0.49 | 0.739 | 0.33 | 0.983 |
| DHAR | 1.55 | 0.202 | 20.58 | 0.000 | 14.56 | 0.000 | 0.54 | 0.882 | 0.05 | 0.985 | 1.35 | 0.251 | 0.37 | 0.971 |
